# Supplementary material for: iMOE: prediction of second-life battery degradation trajectory using interpretable mixture of experts
Source: Nat Commun. 2026 Feb 9;17:2549. doi: 10.1038/s41467-026-69369-1 (PMC13000264; doi:10.1038/s41467-026-69369-1)
Supplement: Supplementary file 1 — Supplementary Information [file 41467_2026_69369_MOESM1_ESM.pdf]

Supplementary Information

iMOE: Prediction of second-life battery degradation trajectory using interpretable mixture of experts

Xinghao Huang (黄星皓) [1, 8], Shengyu Tao (陶晟宇) [1, 2, 3, 8, *], Chen Liang (梁宸) [1], Yining Tang (唐艺宁) [4], Jiawei Chen (陈嘉炜) [5], Junzhe Shi (石浚喆) [2], Yuqi Li (李钰琦) [6], Bizhong Xia (夏必忠) [1, *], Guangmin Zhou (周光敏)[1, *], Xuan Zhang (张璇) [1, 7, *]

[1] Tsinghua Shenzhen International Graduate School, Tsinghua University, Shenzhen, 518055, China

[2] Department of Civil and Environmental Engineering, UC Berkeley, Berkeley, CA, 94720, USA

[3] Department of Electrical Engineering, Chalmers University of Technology, Gothenburg, 41296, Sweden

[4] Civil and Environmental Engineering, Stanford University, Stanford, CA, 94305, USA

[5] School of Computer Science, Peking University, Beijing 100871, China

[6] Department of Materials Science and Engineering, Stanford University, Stanford, CA, 94305, USA

[7] Center of International Innovation for Technology and Science, Shenzhen, Guangdong, 518063, China

[8] These authors contribute equally to this work.

* Corresponding authors:

shengyu.tao@chalmers.se (S. Tao); xiabz@sz.tsinghua.edu.cn (B. Xia),

guangminzhou@sz.tsinghua.edu.cn (G. Zhou); xuanzhang@sz.tsinghua.edu.cn (X. Zhang).

This file contains:

Supplementary Figures 1-30;

Supplementary Tables 1-12;

Supplementary Notes 1-11.

Supplementary Figure 1. QV curves and relaxation voltage curves of NCA material batteries.

In the UL dataset, the QV curves and relaxation voltage curves of NCA material batteries exhibit a declining trend with aging within the selected input data range. As the SOH decreases, both curves show a downward trend, with different colors mapping distinct SOH values, indicating a strong correlation between the selected input data and battery degradation. (a)-(e) represent batteries with different materials, each randomly selecting one cell for visualization in the corresponding plot.

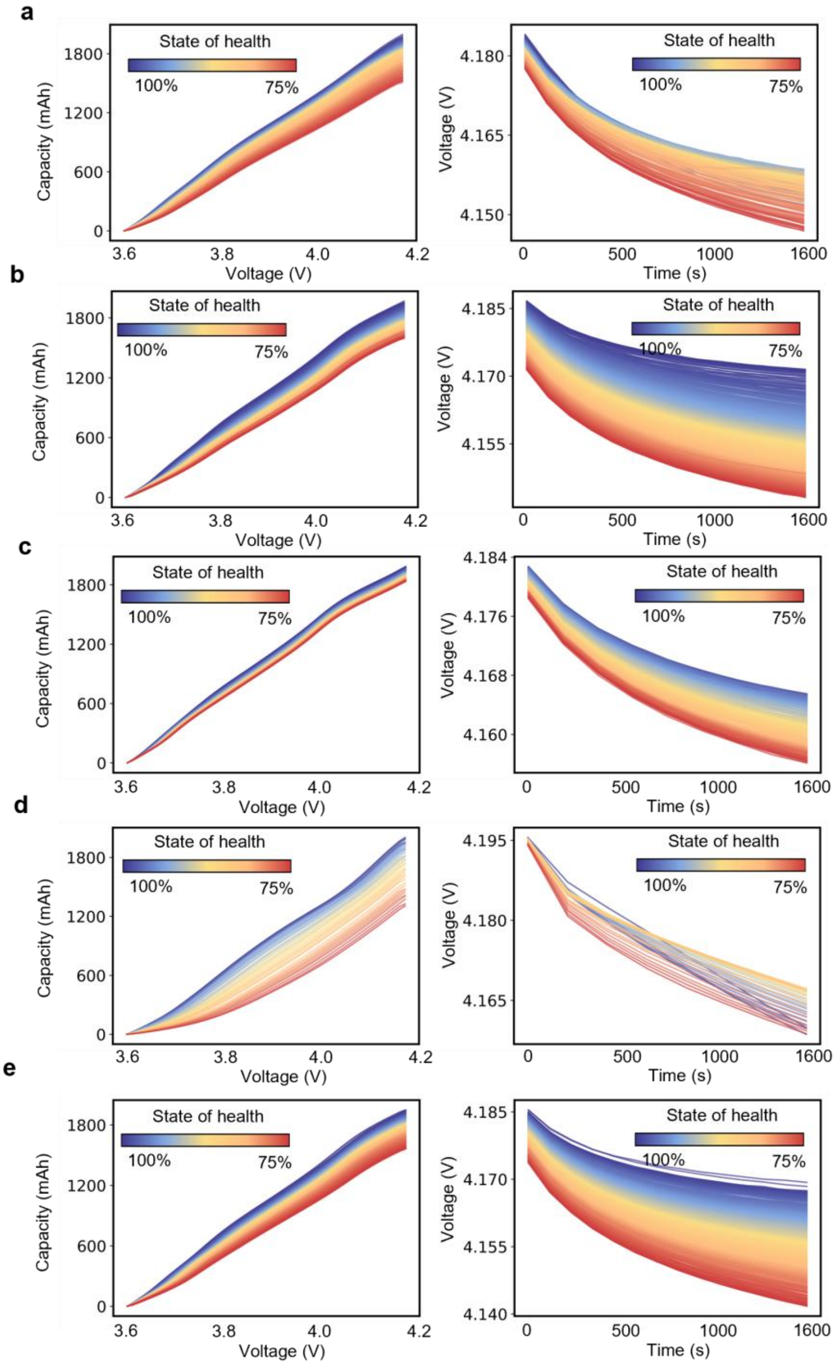

Supplementary Figure 2. QV curves and relaxation voltage curves of NCM material batteries.

In the UL dataset, the QV curves and relaxation voltage curves of NCM material batteries exhibit a declining trend with aging within the selected input data range. As the SOH decreases, both curves show a downward trend, with different colors mapping distinct SOH values, indicating a strong correlation between the selected input data and battery degradation. (a)-(c) represent batteries with different materials, each randomly selecting one cell for visualization in the corresponding plot.

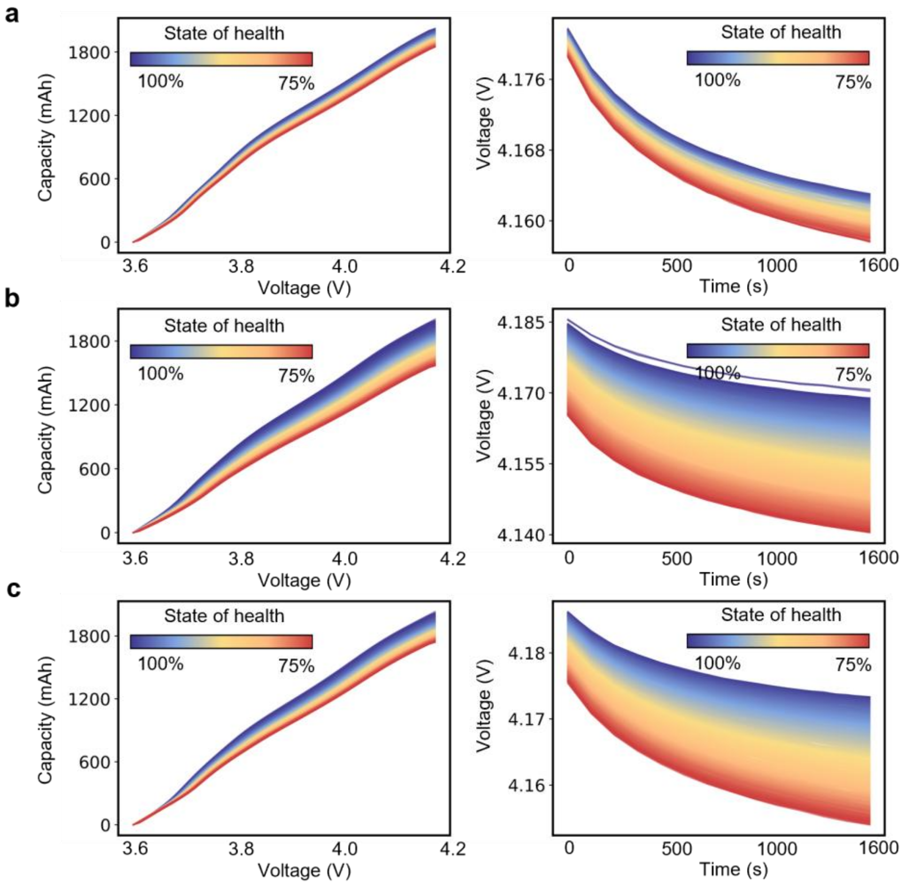

Supplementary Figure 3. QV curves and relaxation voltage curves of NCMNCA material batteries.

In the UL dataset, the QV curves and relaxation voltage curves of NCMNCA material batteries exhibit a declining trend with aging within the selected input data range. As the SOH decreases, both curves show a downward trend, with different colors mapping distinct SOH values, indicating a strong correlation between the selected input data and battery degradation. (a)-(c) represent batteries with different materials, each randomly selecting one cell for visualization in the corresponding plot.

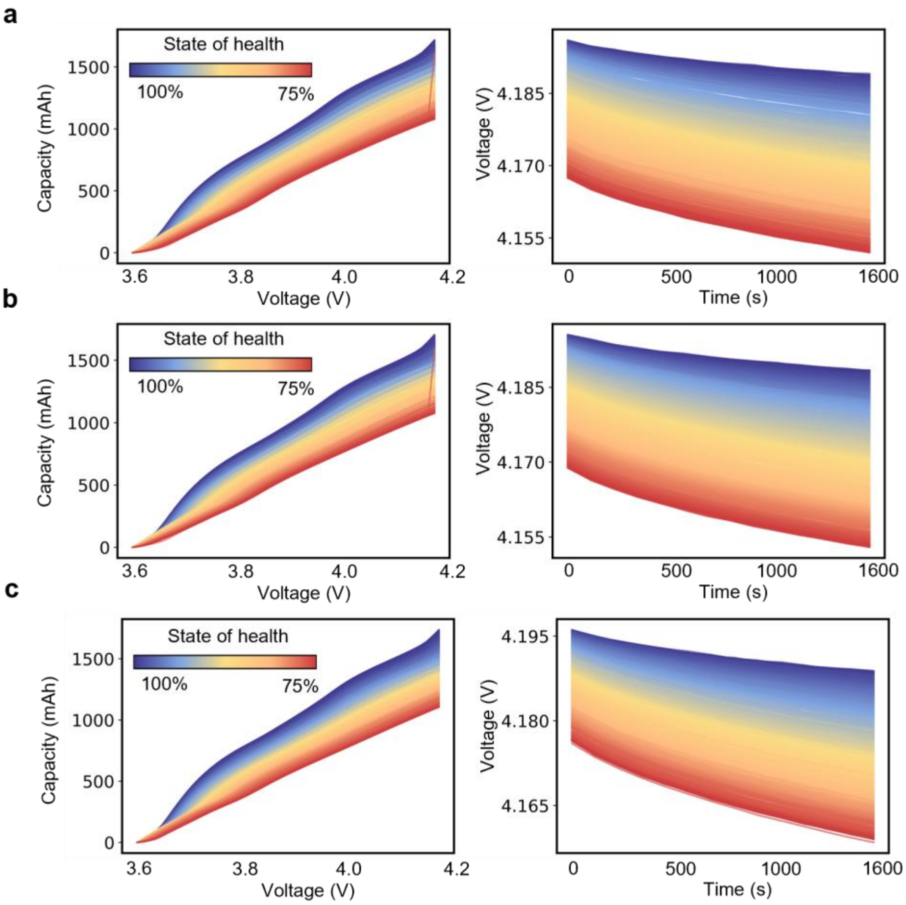

Supplementary Figure 4. 12 features in the NCA material batteries.

Aging trends of 12 features in the NCA material battery dataset, with different colors representing different operating conditions. Among these, six features are extracted from the QV curves, and six are derived from the relaxation voltage curves (specific extraction methods are detailed in Supplementary Note 2). The feature values exhibit nearly linear trends over the aging process, demonstrating a strong correlation between these features and battery degradation.

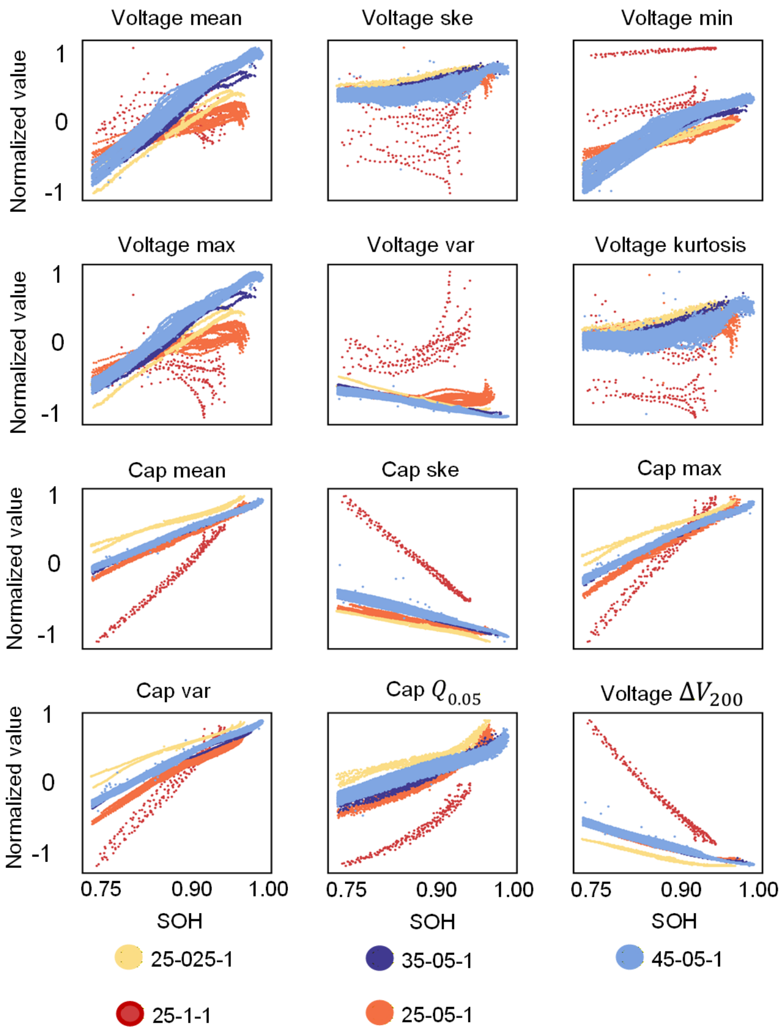

Supplementary Figure 5. 12 features in the NCM material batteries.

Aging trends of 12 features in the NCM material battery dataset, with different colors representing different operating conditions. Among these, six features are extracted from the QV curves, and six are derived from the relaxation voltage curves (specific extraction methods are detailed in Supplementary Note 2). The feature values exhibit nearly linear trends over the aging process, demonstrating a strong correlation between these features and battery degradation.

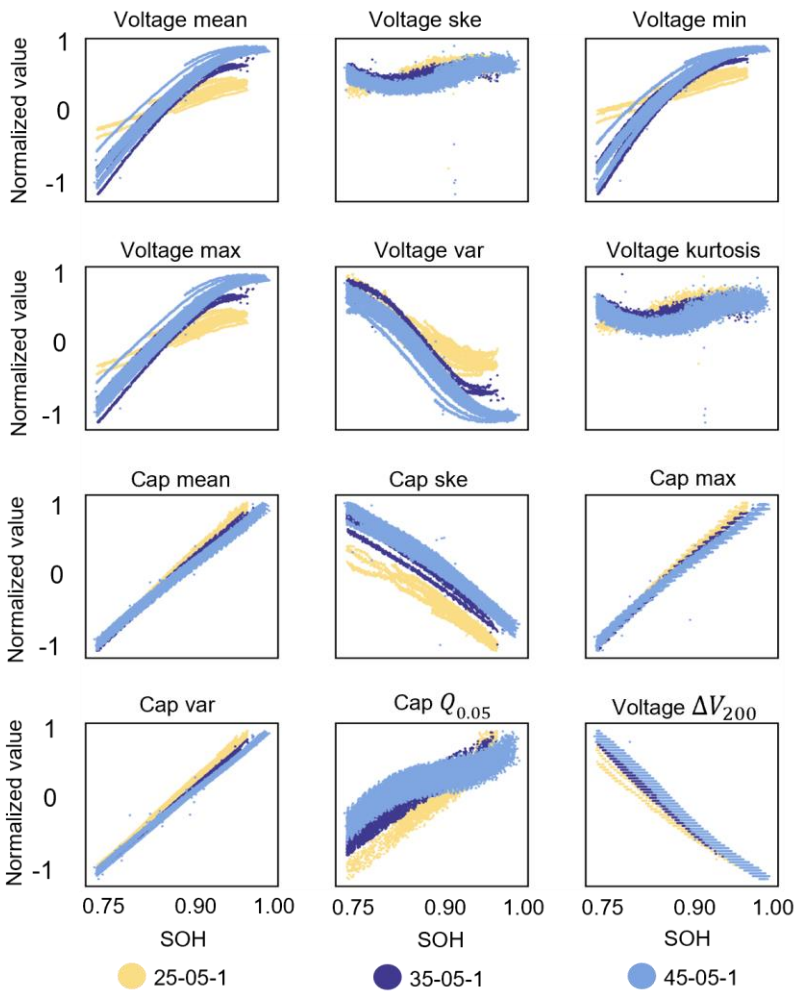

Supplementary Figure 6. 12 features in the NCMNCA material batteries.

Aging trends of 12 features in the NCMNCA material battery dataset, with different colors representing different operating conditions. Among these, six features are extracted from the QV curves, and six are derived from the relaxation voltage curves (specific extraction methods are detailed in Supplementary Note 2). The feature values exhibit nearly linear trends over the aging process, demonstrating a strong correlation between these features and battery degradation.

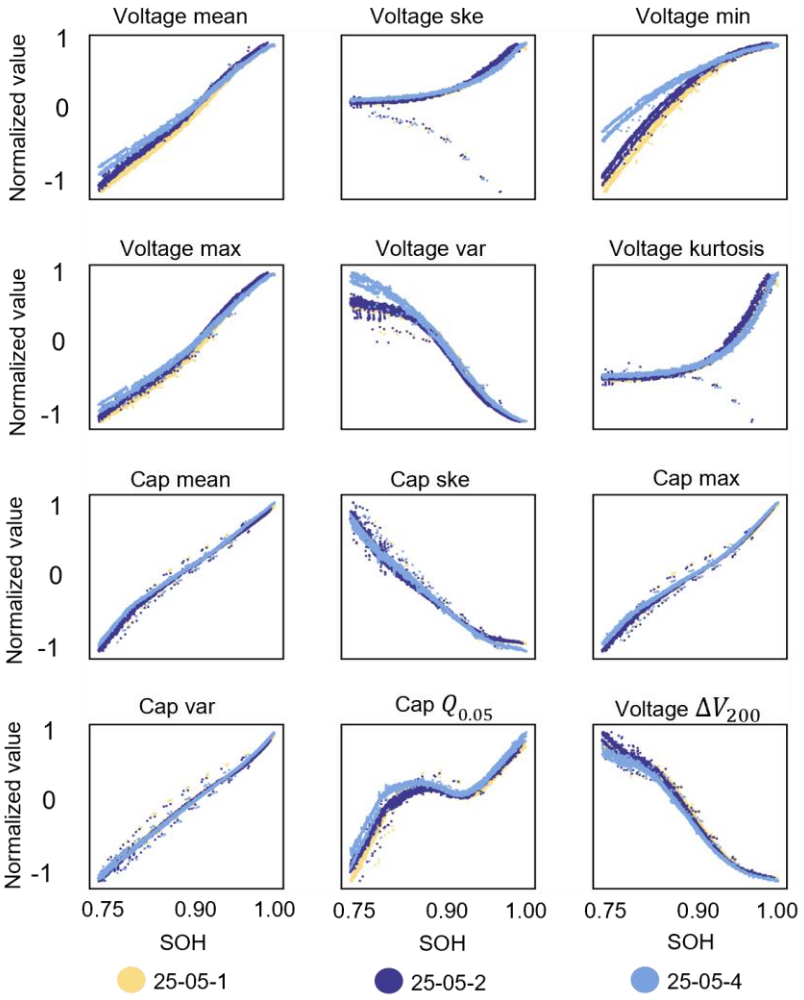

Supplementary Figure 7. Machine learning pipeline design of the iMOE.

The initial input consists of randomly sampled SOC data collected from battery field operations, including capacity-voltage curves and relaxation voltage curves. After feature engineering and standardization, these curves are fed into the degradation router. The router outputs weights corresponding to different expert networks. The original capacity-voltage curves are adaptively resampled, and based on the expert network weights, the predictions from multiple experts are synthesized to output degradation trends. These degradation trends are then cyclically combined with future operating condition predictions to estimate degradation trajectories under uncertain conditions. The loss function incorporates both $Loss_{cv}$ (calculated based on the importance weights of samples from different degradation stages within a batch) and $Loss_{MSE}$ (measuring the discrepancy between predicted and actual values). In the figure, **N** represents the number of experts.

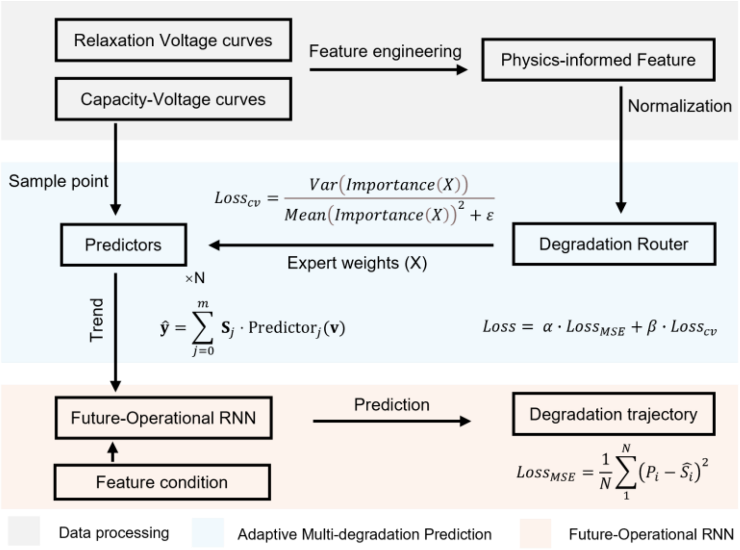

Supplementary Figure 8. IC Aging Curve Analysis of the Dataset

This section aims to demonstrate the rationality and universality of dividing battery aging into multiple stages using IC curves, and to explain how these parameters reveal the battery degradation mechanisms and reflect their physical significance. This assumption is based on numerous studies on lithium battery degradation patterns. For instance, existing research suggests that during the battery's lifecycle, the formation and thickening of the SEI layer affect the capacity degradation in the early stages, while lithium-ion deposition typically occurs in the later stages of battery use and is one of the main causes of the sharp capacity decline.

IC curves reveal internal changes in the battery by showing the subtle variations between battery capacity and voltage, especially during the aging process, where the SEI layer growth, lithium-ion loss, and internal electrochemical reactions significantly impact the IC curve. As shown in the figure, different colors represent different SOH samples. In the early charging and discharging cycles, the curve displays sharp peaks, indicating the battery is in a healthy state with significant and rapid capacity changes, mainly reflecting the formation of the SEI layer and surface chemical reactions. As the cycle count increases, the peaks of the IC curve become more rounded and shift to the right, and longer flat sections appear, indicating that the battery's reversible capacity is gradually decreasing. When the battery enters a more severe degradation phase, the peaks of the IC curve gradually decrease. At this point, degradation mechanisms such as lithium-ion deposition and loss of active materials dominate the capacity decline. The widening of the peaks in the curve and the sharp capacity drop indicate that the battery's energy storage capacity is severely compromised.

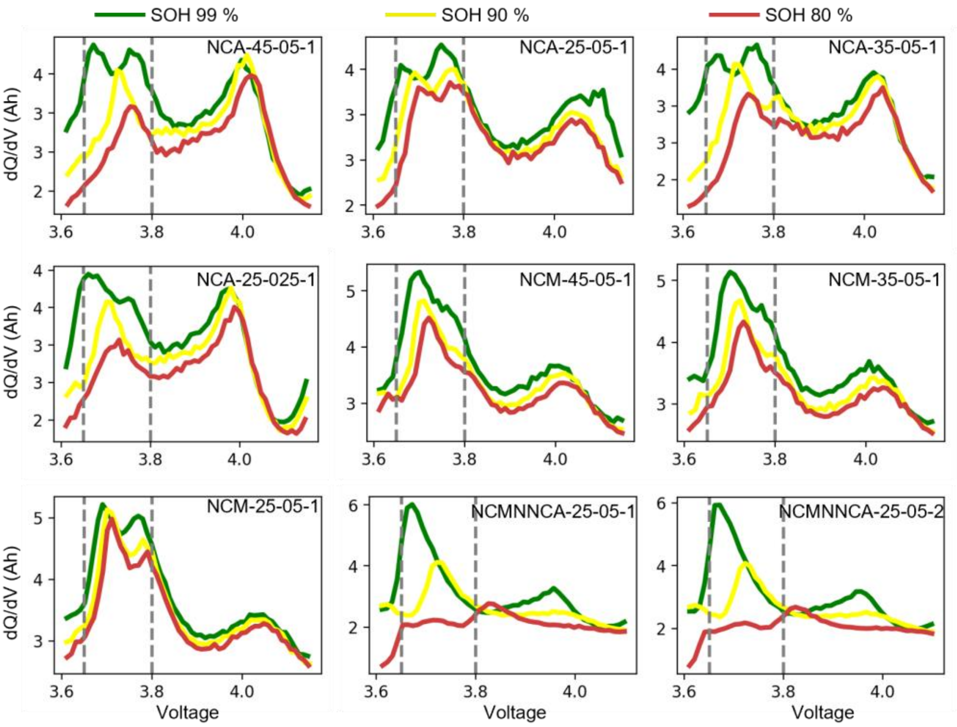

Supplementary Figure 9. Prediction Results of Full Lifecycle Health Status for NCA Material Batteries.

Predicting the degradation trajectory of NCA material batteries for arbitrary samples throughout their full lifecycle. A sliding window approach is employed for data extraction and prediction (details provided in Supplementary Note 1). (a) presents the prediction results under the 25-1-1 operating condition, where the typical lifespan is approximately 30 cycles. Accordingly, predictions are made for the next 10 cycles based on the current cycle. (b)-(e) respectively display prediction results under different operating conditions, where only partial data from the current cycle is used to forecast the next 50 cycles for individual batteries. The color gradient represents the health states of different samples. (Naming conventions are specified in Supplementary Table 1).

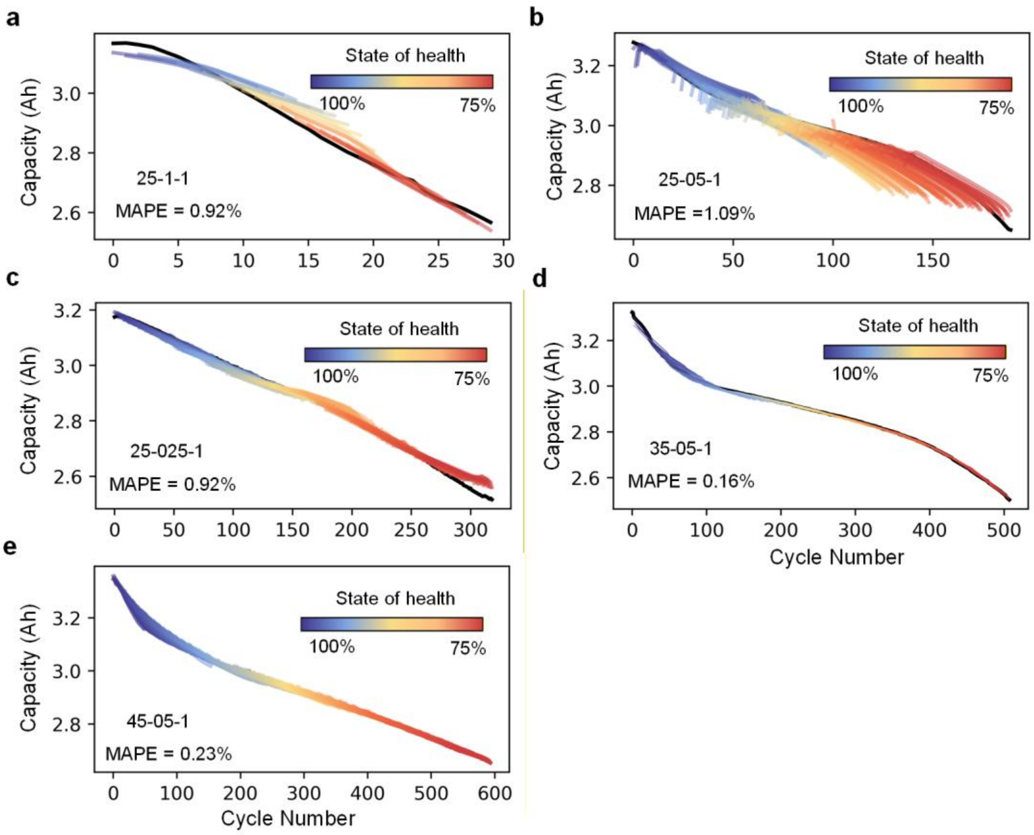

Supplementary Figure 10. Prediction Results of Full Lifecycle Health Status for NCM Material Batteries.

Predicting the degradation trajectory of arbitrary NCM material battery samples throughout their full lifecycle. Using a sliding window approach for data extraction and prediction (see Supplementary Note 1 for details), the method predicts the next 50 cycles for individual batteries based solely on partial data from the current cycle. (a)-(c) present prediction results under different operating conditions, with color coding representing the health states of different samples. (Naming conventions are specified in Supplementary Table 1).

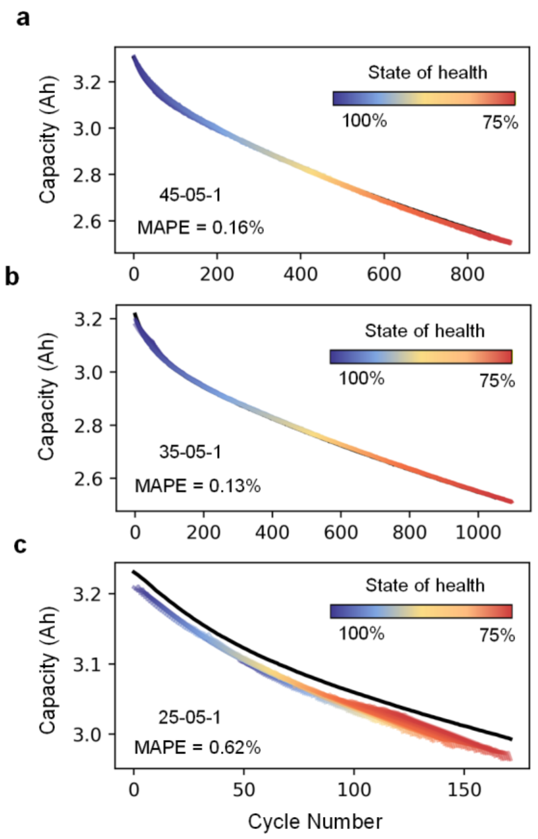

Supplementary Figure 11. Prediction Results of Full Lifecycle Health Status for NCMNCA Material Batteries.

Predicting the degradation trajectory of arbitrary NCMNCA material battery samples throughout their full lifecycle. Using a sliding window approach for data extraction and prediction (see Supplementary Note 1 for details), the method predicts the next 50 cycles for individual batteries based solely on partial data from the current cycle. (a)-(c) present prediction results under different operating conditions, with color coding representing the health states of different samples. (Naming conventions are specified in Supplementary Table1).

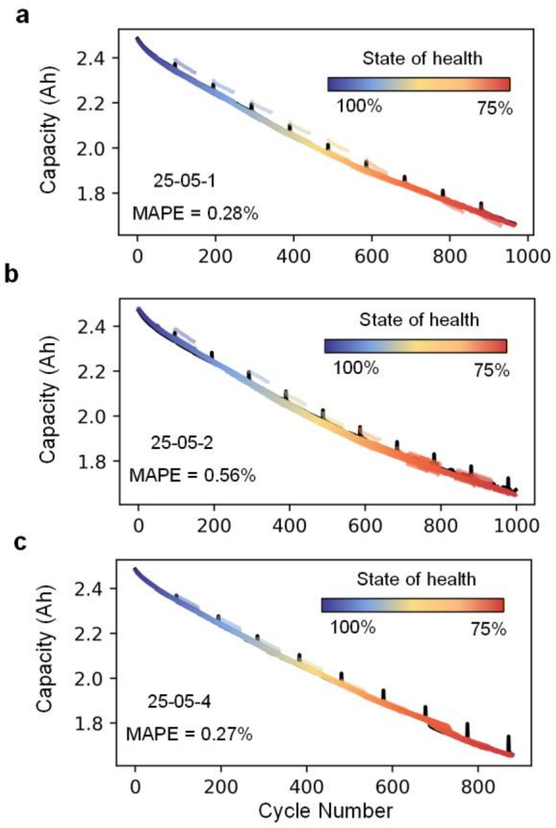

Supplementary Figure 12. Prediction Results of Full Lifecycle Health Status for TPSL-Arbitrary load-conditioned batteries.

Predicting degradation trajectories under repurposed load-varying conditions. For TPSL-Arbitrary load-conditioned batteries, the method employs a sliding window approach for data extraction and prediction (detailed in Supplementary Note 1). Using only partial data from the current cycle, it predicts the next 50 cycles for individual batteries, where red points represent predicted values and black points denote the original capacity degradation curves. (a)-(c) display prediction results for different samples, showing iMOE's accurate prediction of both battery capacity degradation and capacity variations during load condition changes.

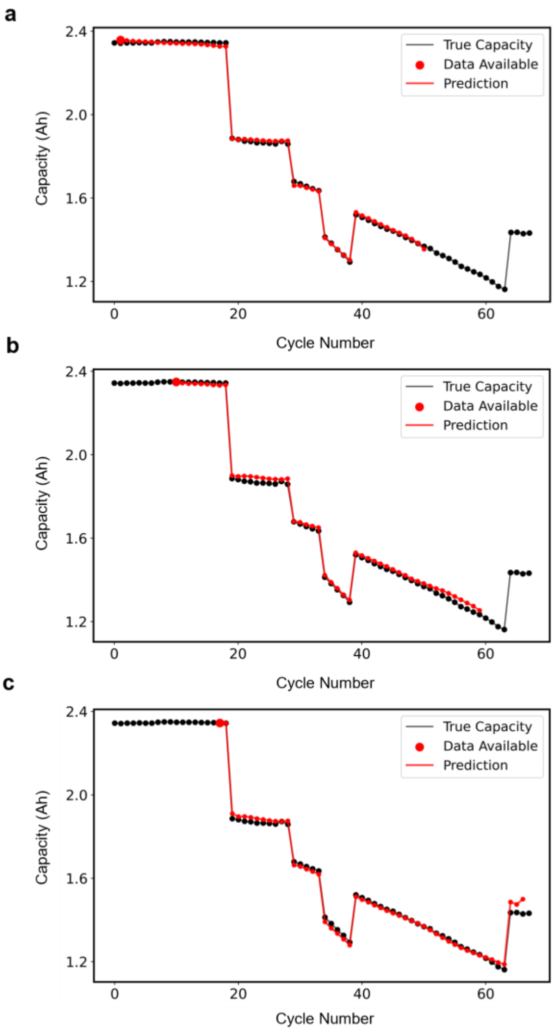

Supplementary Figure 13. Prediction Results of Full Lifecycle Health Status for TPSL- Fixed load-conditioned batteries.

Predicting degradation trajectories under repurposed load-varying conditions. For TPSL-Fixed load-conditioned batteries, the method employs a sliding window approach for data extraction and prediction (detailed in Supplementary Note 1). Using only partial data from the current cycle, it predicts the next 50 cycles for individual batteries, where red points represent predicted values and black points denote the original capacity degradation curves. (a)-(c) display prediction results for different samples, showing iMOE's accurate prediction of both battery capacity degradation and capacity variations during load condition changes.

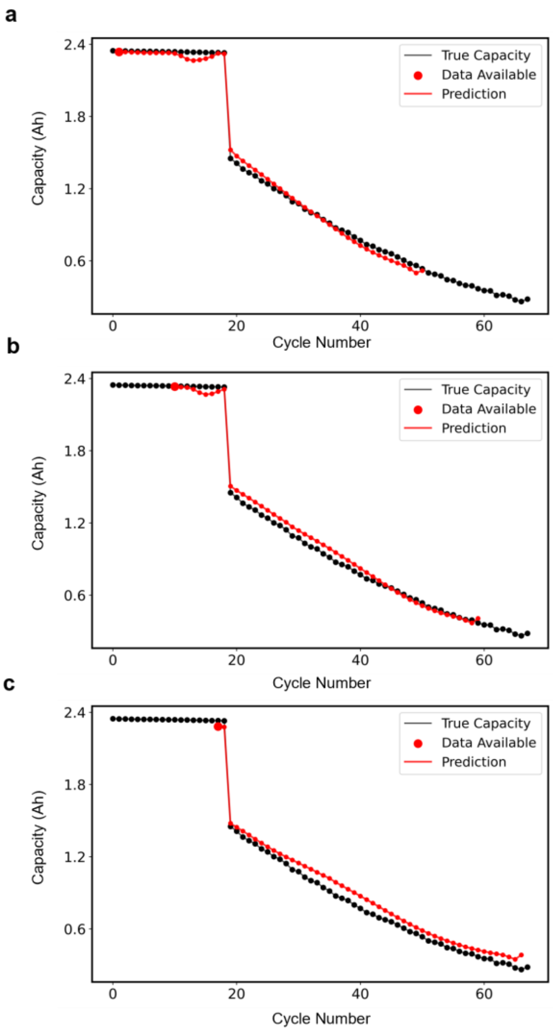

Supplementary Figure 14. Prediction Results of Full Lifecycle Health Status for TPSL-Arbitrary load-conditioned batteries.

Predicting degradation trajectories under repurposed load-varying conditions. For TPSL-Fixed load-conditioned batteries, the method employs a sliding window approach for data extraction and prediction (detailed in Supplementary Note 1). Using only partial data from the current cycle, it predicts the next 50 cycles for individual batteries, where red points represent predicted values and black points denote the original capacity degradation curves. (a)-(c) display prediction results for different samples, showing iMOE's accurate prediction of both battery capacity degradation and capacity variations during load condition changes.

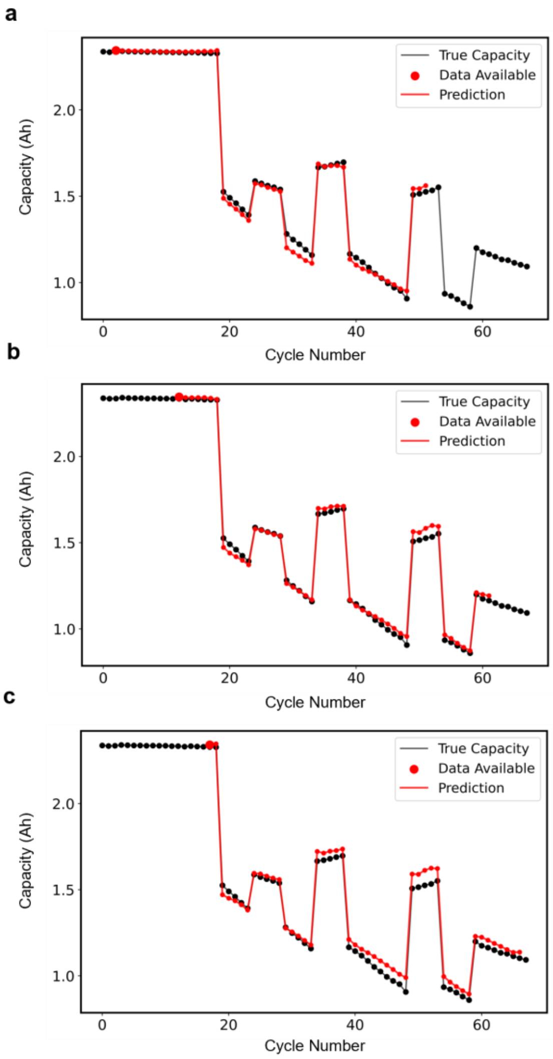

Supplementary Figure 15. Prediction Results of Full Lifecycle Health Status for TPSL- Fixed load-conditioned batteries.

Predicting degradation trajectories under repurposed load-varying conditions. For TPSL-Fixed load-conditioned batteries, the method employs a sliding window approach for data extraction and prediction (detailed in Supplementary Note 1). Using only partial data from the current cycle, it predicts the next 50 cycles for individual batteries, where red points represent predicted values and black points denote the original capacity degradation curves. (a)-(c) display prediction results for different samples, showing iMOE's accurate prediction of both battery capacity degradation and capacity variations during load condition changes.

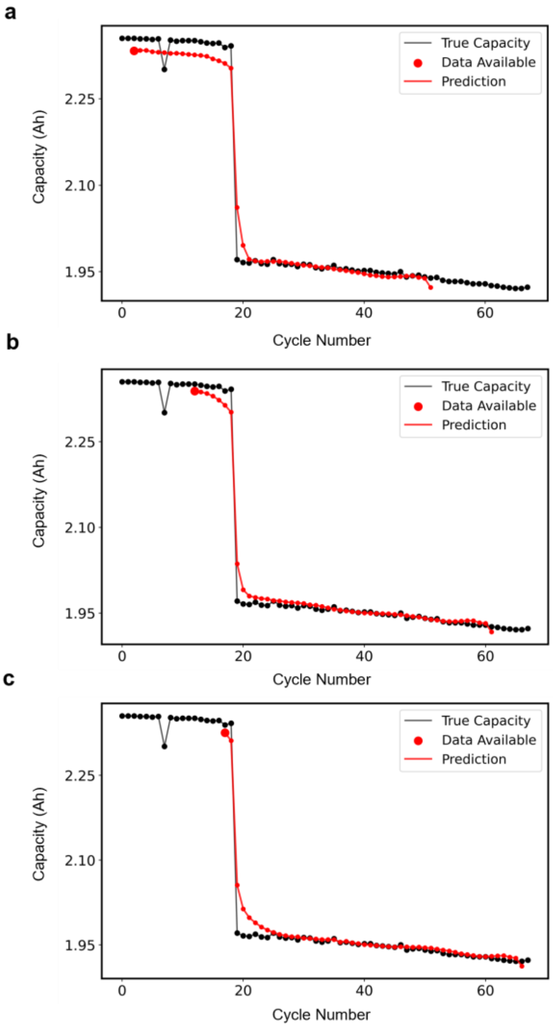

Supplementary Figure 16. Correlation between expert weights and degradation stages under different operating conditions.

Correlation between expert weights and degradation stages under different operating conditions. By randomly selecting pre-trained models corresponding to specific conditions and using single-cycle test samples from the full lifecycle of batteries across different datasets as input, we visualize the relationship between the evolving expert weights in the model output and the degradation stages. The horizontal axis represents test samples from different degradation stages, while the vertical axis indicates the weights assigned by five experts to each sample, with color intensity reflecting the magnitude of expert weights. As degradation progresses, different experts dominate corresponding stages, enabling adaptive integration of expert weights to predict degradation trajectories for samples at various degradation phases.

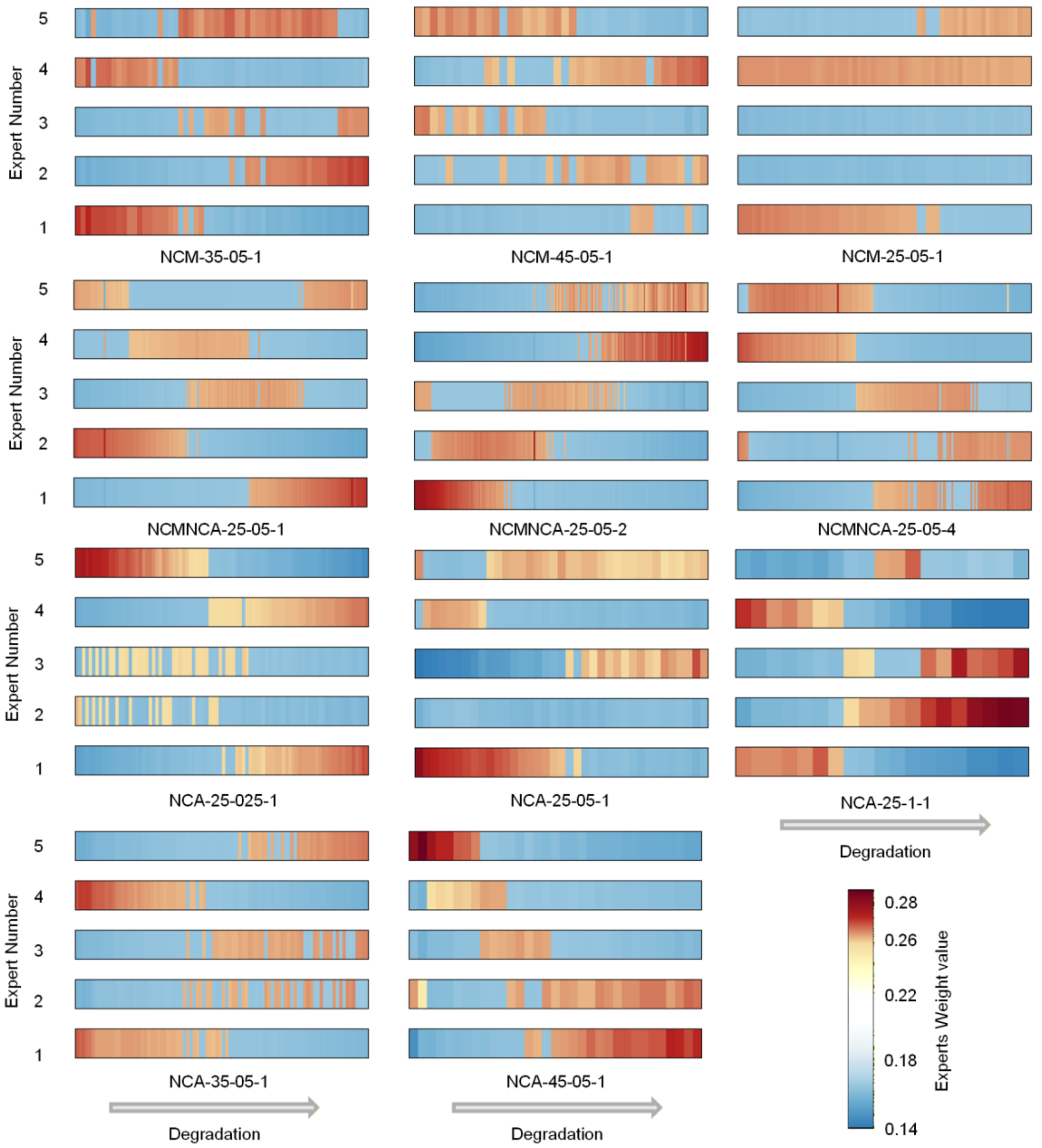

Supplementary Figure 17. t-SNE Dimensionality Reduction Visualization of Expert Weights from NCA Battery Test Model.

For the NCA-material test batteries under different operating conditions in the UL dataset, we randomly selected a pre-trained model and used full-lifecycle single-cycle test samples as input. After t-SNE clustering, the expert weights output by the model were visualized, with samples from different aging stages color-mapped accordingly (see Supplementary Note 5 for details). In Supplementary Figs. 1-6, as well as through SHAP and Spearman correlation coefficient analysis, the features input into the AMDP module show strong correlation with the degradation stages themselves. In the two-dimensional latent space after clustering, there is a clear boundary between healthy and aged batteries, further demonstrating the effectiveness of the MOE architecture in identifying different degradation stages and validating the rationale for using expert weights in second-life utilization decisions. Figures (a)-(d) represent three different operating conditions.

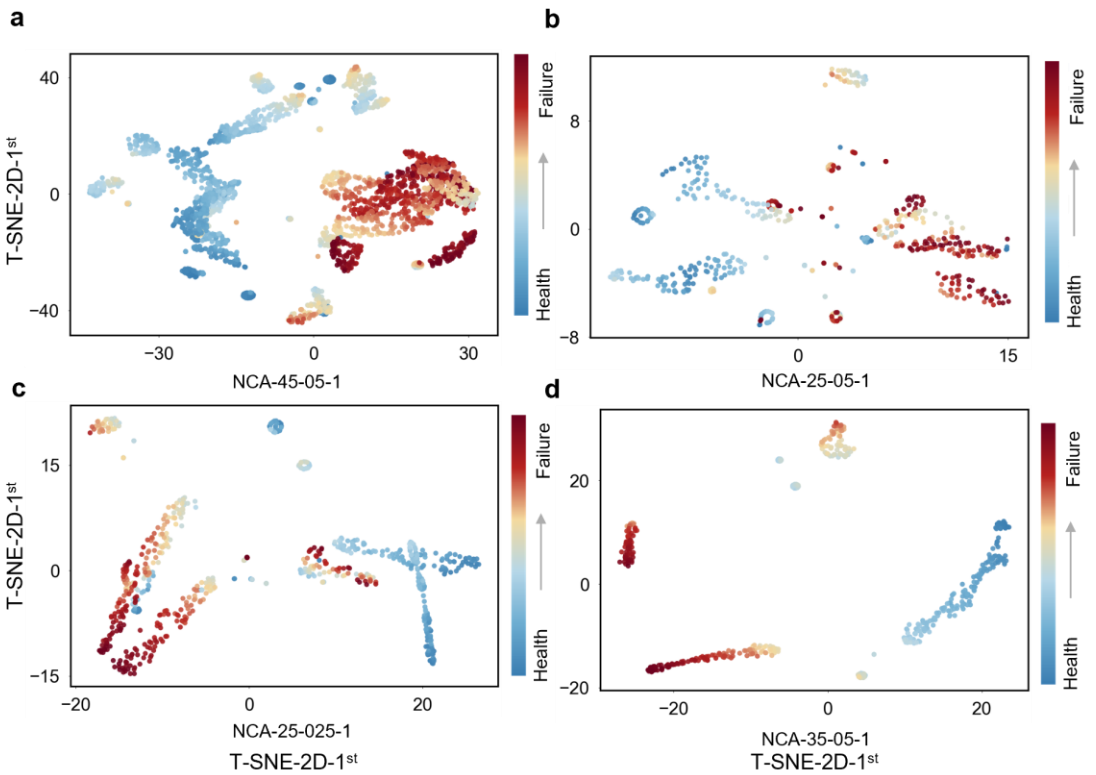

Supplementary Figure 18. t-SNE Dimensionality Reduction Visualization of Expert Weights from NCM Battery Test Model.

For the NCM-material test batteries under different operating conditions in the UL dataset, we randomly selected a pre-trained model and used full-lifecycle single-cycle test samples as input. After t-SNE clustering, the expert weights output by the model were visualized, with samples from different aging stages color-mapped accordingly (see Supplementary Note 5 for details). In Supplementary Figs. 1-6, as well as through SHAP and Spearman correlation coefficient analysis, the features input into the AMDP module show strong correlation with the degradation stages themselves. In the two-dimensional latent space after clustering, there is a clear boundary between healthy and aged batteries, further demonstrating the effectiveness of the MOE architecture in identifying different degradation stages and validating the rationale for using expert weights in second-life utilization decisions. Figures (a)-(c) represent three different operating conditions.

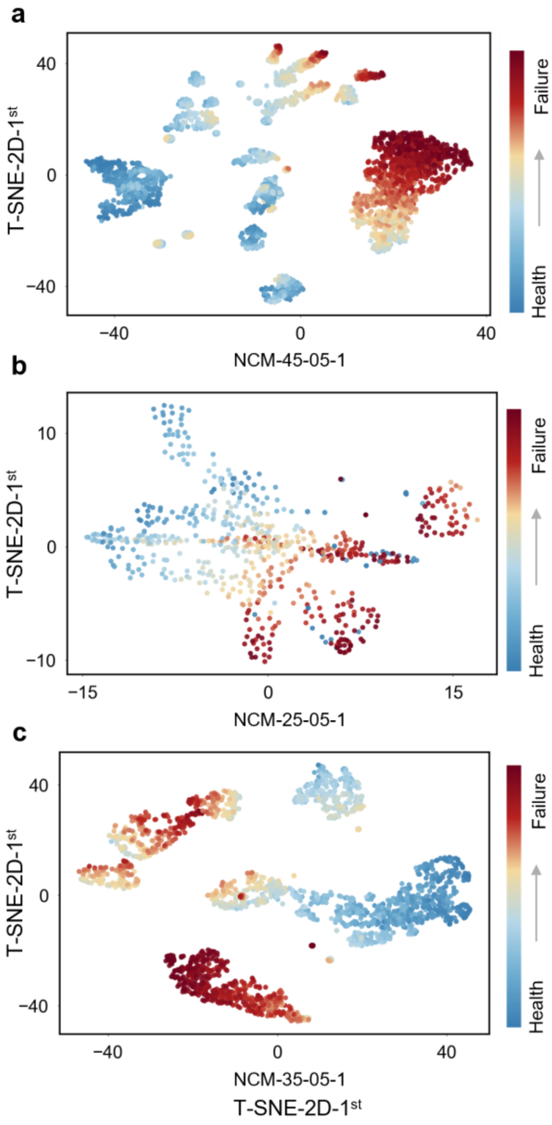

Supplementary Figure 19. t-SNE Dimensionality Reduction Visualization of Expert Weights from NCMNCA Battery Test Model.

For the NCMNCA-material test batteries under different operating conditions in the UL dataset, we randomly selected a pre-trained model and used full-lifecycle single-cycle test samples as input. After t-SNE clustering, the expert weights output by the model were visualized, with samples from different aging stages color-mapped accordingly (see Supplementary Note 5 for details). In Supplementary Figs. 1-6, as well as through SHAP and Spearman correlation coefficient analysis, the features input into the AMDP module show strong correlation with the degradation stages themselves. In the two-dimensional latent space after clustering, there is a clear boundary between healthy and aged batteries, further demonstrating the effectiveness of the MOE architecture in identifying different degradation stages and validating the rationale for using expert weights in second-life utilization decisions. Figures (a)-(c) represent three different operating conditions.

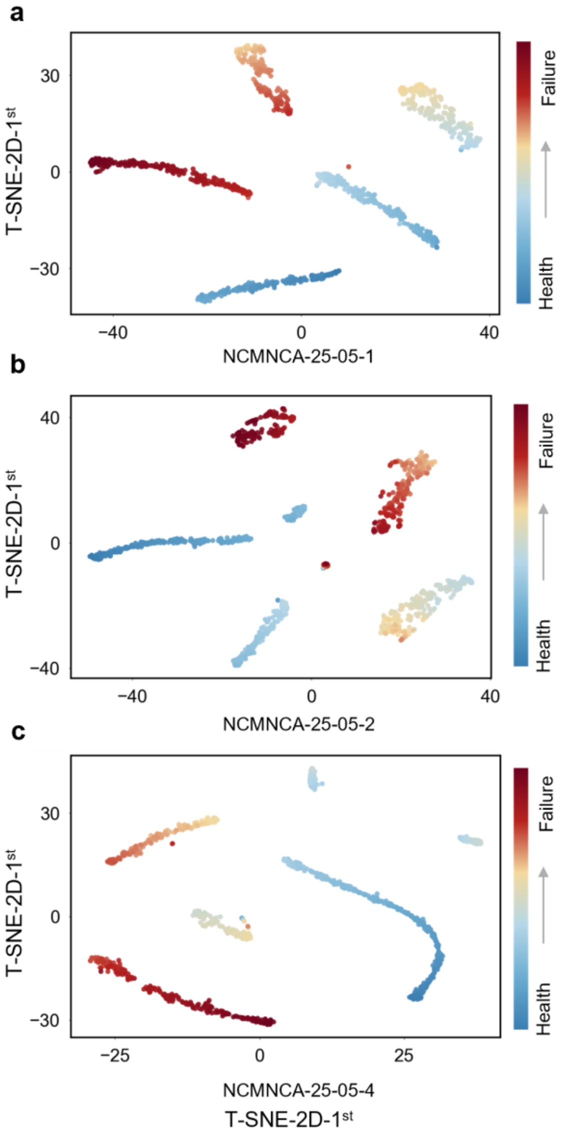

Supplementary Figure 20. Expert weights output by the model for an entire batch of NCM-material batteries retired at different SOH levels.

Visualization of expert weights output by the model for an entire batch of batteries retired at different SOH levels during practical deployment. For all NCM-material batteries in selected SOH retirement scenarios, we randomly chose one pre-trained model to visualize the expert weights across the entire battery population. The horizontal axis represents the number of retired batteries, while the vertical axis indicates the weights assigned by five corresponding experts, with color intensity reflecting weight values Fig. (a), (b), and (c) represent three distinct operating conditions: 25-05-1, 35-05-1, and 45-05-1 respectively.

The model enables optimal utilization decisions by processing partial cycle data obtained from retired batteries as input and generating interpretable expert weight outputs. In certain datasets, the number of batteries at 95% SOH differs from those at 75% SOH, as some batteries were retired before reaching 75% SOH, resulting in a reduced test population. Nevertheless, the model achieves highly accurate and interpretable classification results for battery samples at selected SOH levels (see Supplementary Note 6 for details).

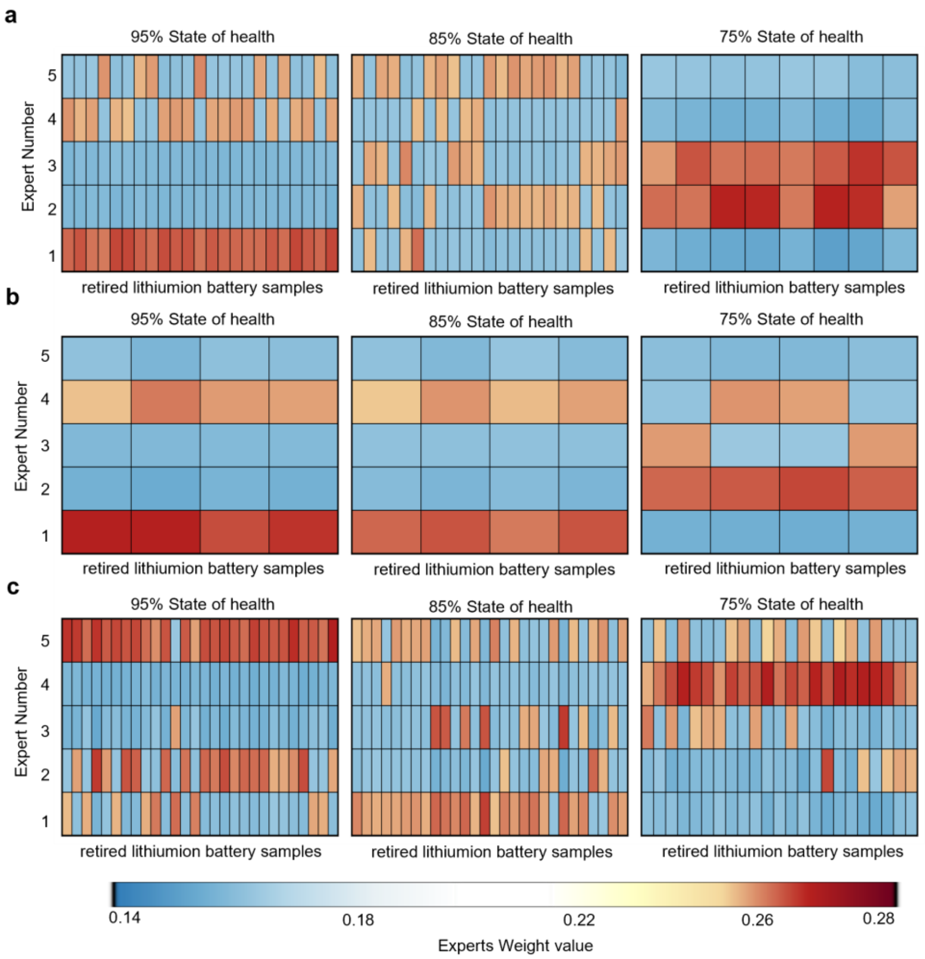

Supplementary Figure 21. Expert weights output by the model for an entire batch of NCMNCA-material batteries retired at different SOH levels.

Visualization of expert weights output by the model for an entire batch of batteries retired at different SOH levels during practical deployment. For all NCMNCA-material batteries in selected SOH retirement scenarios, we randomly chose one pre-trained model to visualize the expert weights across the entire battery population. The horizontal axis represents the number of retired batteries, while the vertical axis indicates the weights assigned by five corresponding experts, with color intensity reflecting weight values, Fig. (a), (b), and (c) represent three distinct operating conditions: 25-05-1, 25-05-2, and 25-05-4 respectively.

The model enables optimal utilization decisions by processing partial cycle data obtained from retired batteries as input and generating interpretable expert weight outputs. In certain datasets, the number of batteries at 95% SOH differs from those at 75% SOH, as some batteries were retired before reaching 75% SOH, resulting in a reduced test population. Nevertheless, the model achieves highly accurate and interpretable classification results for battery samples at selected SOH levels (see Supplementary Note 6 for details).

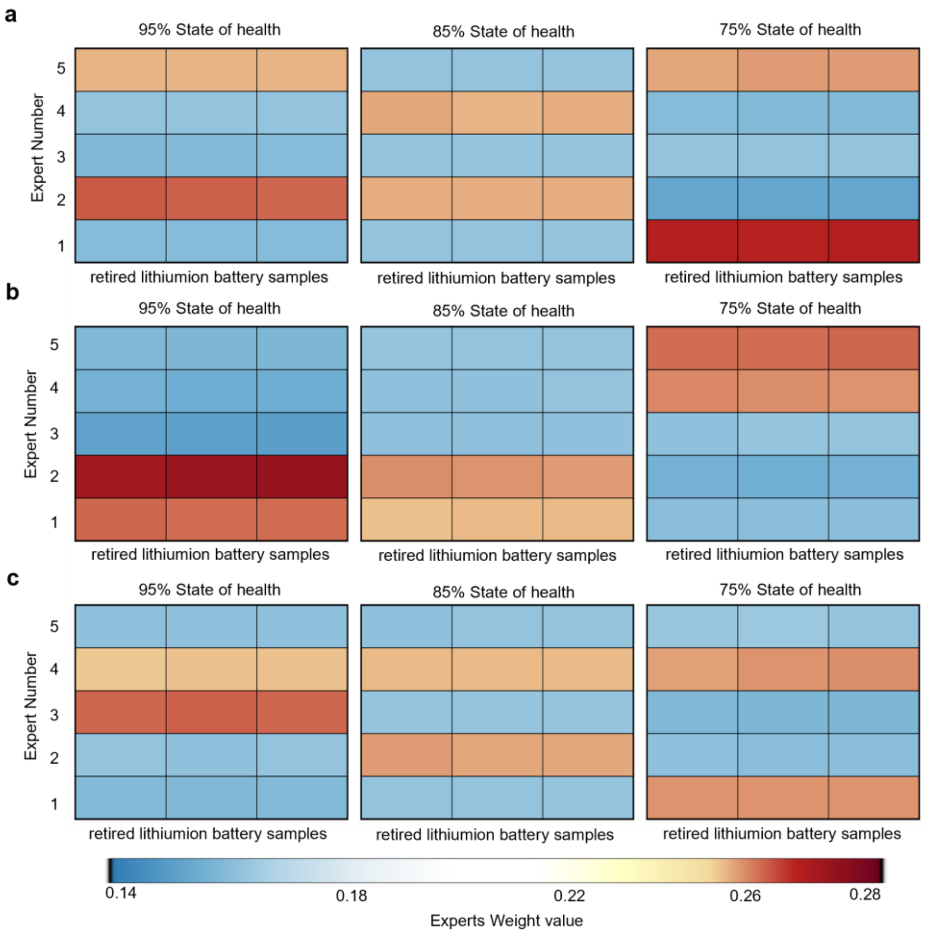

Supplementary Figure 22. Expert weights output by the model for an entire batch of NCA-material batteries retired at different SOH levels.

Visualization of expert weights output by the model for an entire batch of batteries retired at different SOH levels during practical deployment. For all NCA-material batteries in selected SOH retirement scenarios, we randomly chose one pre-trained model to visualize the expert weights across the entire battery population. The horizontal axis represents the number of retired batteries, while the vertical axis indicates the weights assigned by five corresponding experts, with color intensity reflecting weight values Fig. (a)-(e) represent three distinct operating conditions: 25-1-1,25-05-1,25-025-1,35-05-1,45-05-1 respectively.

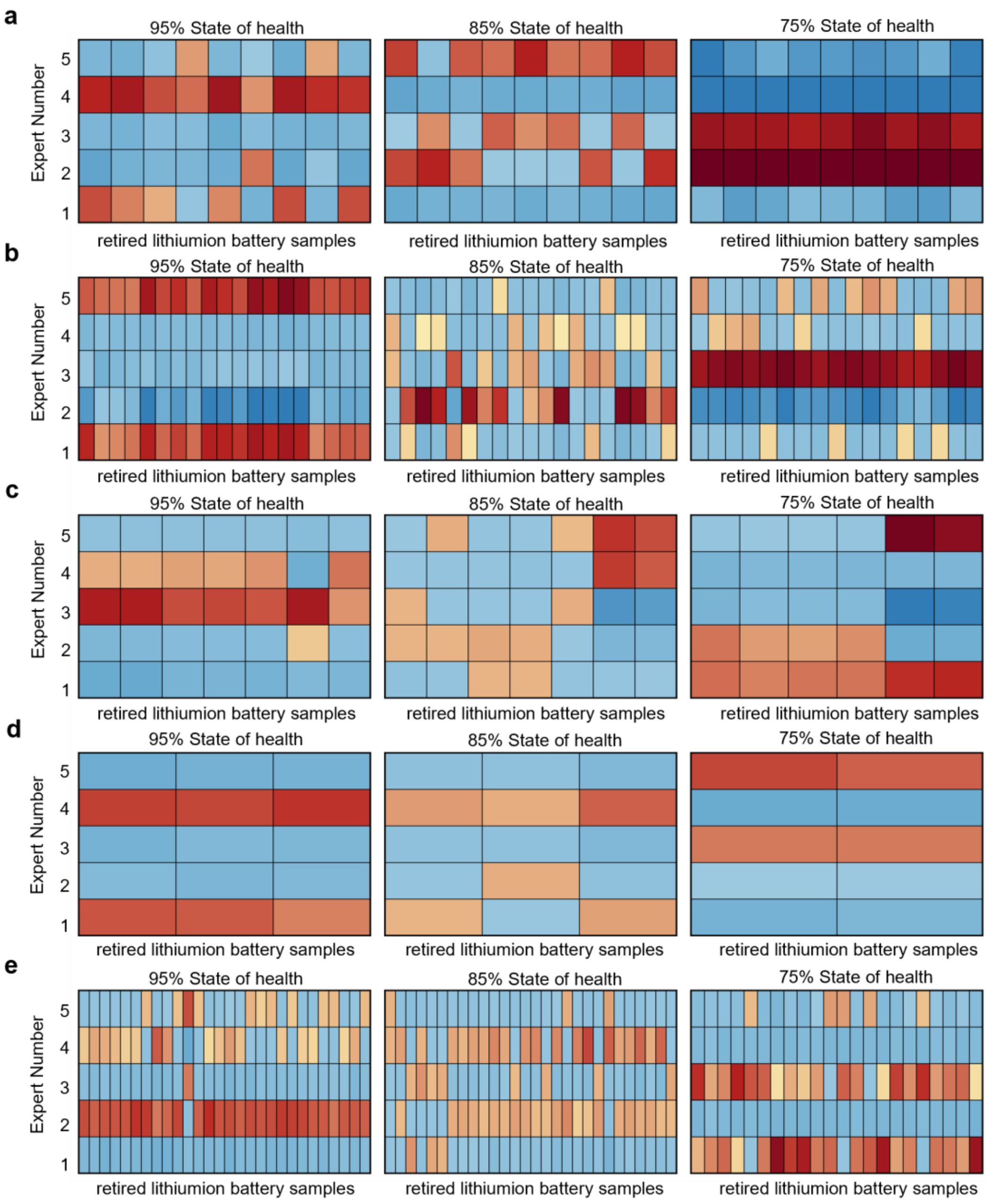

Supplementary Figure 23. Analysis of the Impact of Sensor Noise and Missing Values on Results

In real-world applications, sensor sampling errors are inevitable. This section aims to investigate the impact of sensor sampling errors on estimation results. Specifically, we applied three levels of random Gaussian noise to partial charging curves and relaxation voltages extracted from the field. As shown in Figure (a), different colors represent varying magnitudes of Gaussian noise. On the selected dataset, as the sampling error increases, the model's error also increases linearly.

In extreme cases, incomplete data sampling or missing values may occur. We also considered the impact of such scenarios on the results. Specifically, we randomly removed 20%, 40% of the sampling points, with missing values handled using simple linear interpolation. As shown in Fig. (b), different colors represent different levels of missing data. On the selected dataset, as the amount of missing data increases, the model's error also rises.

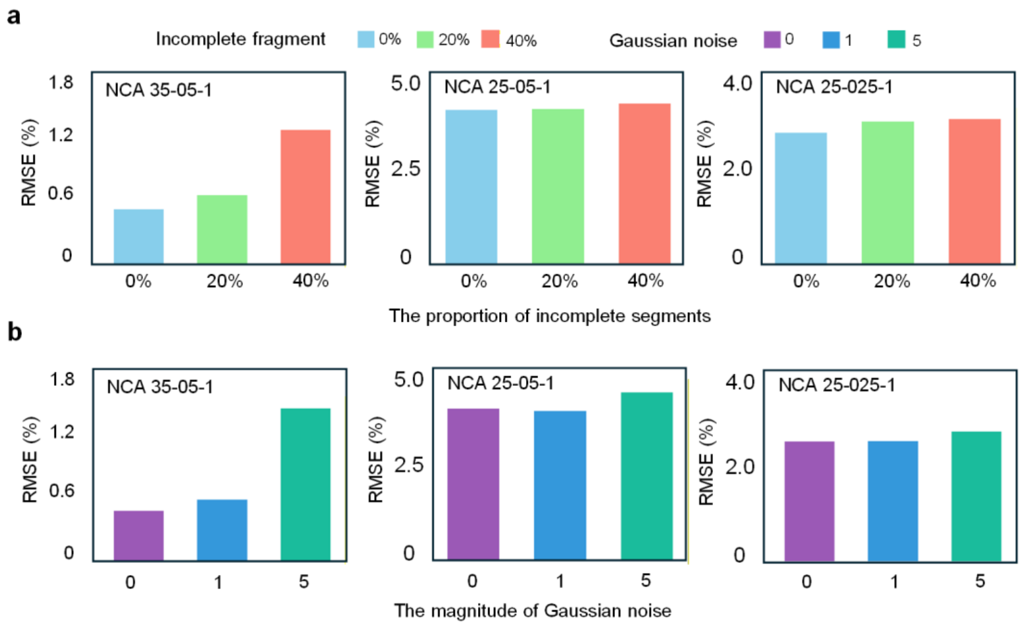

Supplementary Figure 24. Impact of Different Physics-informed Feature on Model Performance.

Here we present the performance changes of the model when reducing physics-informed features. "Both" denotes the complete model incorporating both capacity-voltage curves and relaxation-voltage curves. "no QV features" indicates the removal of six features extracted from capacity-voltage curves, while "no RV features" represents the exclusion of six features derived from relaxation-voltage curves. The results show that removing RV features leads to relatively greater performance degradation, suggesting that relaxation-voltage features may more effectively guide the degradation routing. Nevertheless, the model maintains relatively stable performance overall, demonstrating that the proposed iMOE does not depend on any single specific feature to achieve good performance, though multi-dimensional physics-informed features generally yield better results.

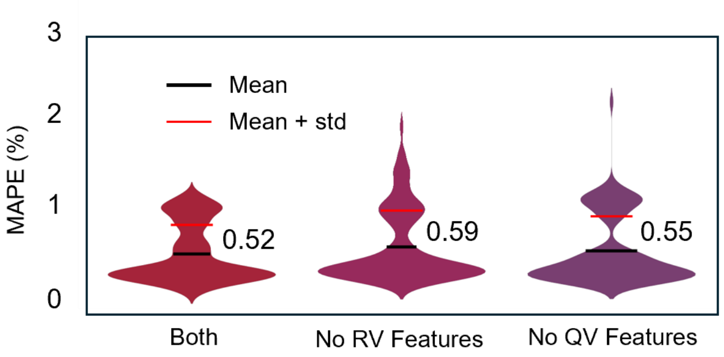

Supplementary Figure 25. Sensitivity Analysis of Physics-informed Feature Noise Across Batteries with Divergent Degradation Paths.

To investigate feature importance, we introduced random Gaussian noise to each extracted feature from raw data, thereby influencing the weight generation for degradation routing in the iMOE model. Fig.(a) demonstrates the performance variations of all 12 features under noise perturbation, with the red baseline indicating the original noise-free model performance. Each experiment was repeated 10 times to ensure reliability. Fig.(b) displays the relationship between predicted and true values across full lifecycle samples under normal conditions, while Fig.(c) shows this relationship for the noise-sensitive feature (Cap mean). The results reveal significant performance degradation when samples were incorrectly assigned to expert networks, further validating the effectiveness of iMOE's two-stage approach that first distinguishes aging phases before predicting degradation trajectories.

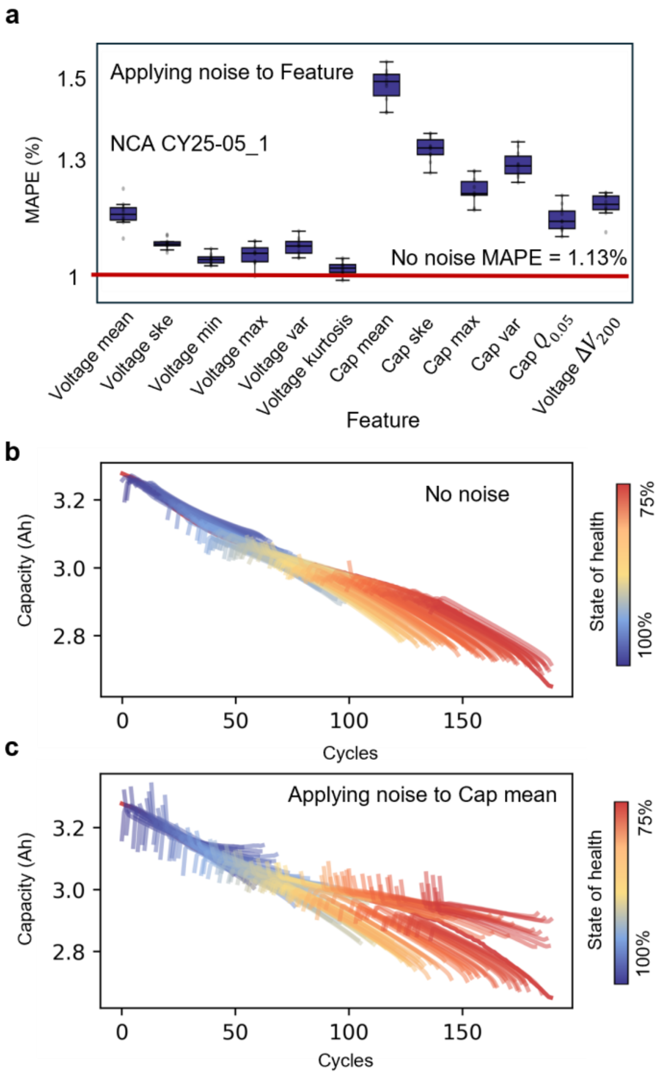

Supplementary Figure 26. Sensitivity Analysis of Physics-informed Feature Noise Across Batteries with Similar Degradation Paths.

To investigate feature importance, we introduced random Gaussian noise to each extracted feature from raw data, thereby influencing the weight generation for degradation routing in the iMOE model. Fig.(a) demonstrates the performance variations of all 12 features under noise perturbation, with the red baseline indicating the original noise-free model performance. Each experiment was repeated 10 times to ensure reliability. Fig.(b) displays the relationship between predicted and true values across full lifecycle samples under normal conditions, while Fig.(c) shows this relationship for the noise-sensitive feature (Voltage max). The results show that even when samples were incorrectly assigned to expert networks, the model performance did not degrade significantly. This observation may be attributed to the inherently small differences in degradation paths among these aged batteries, where different experts performed similar prediction tasks.

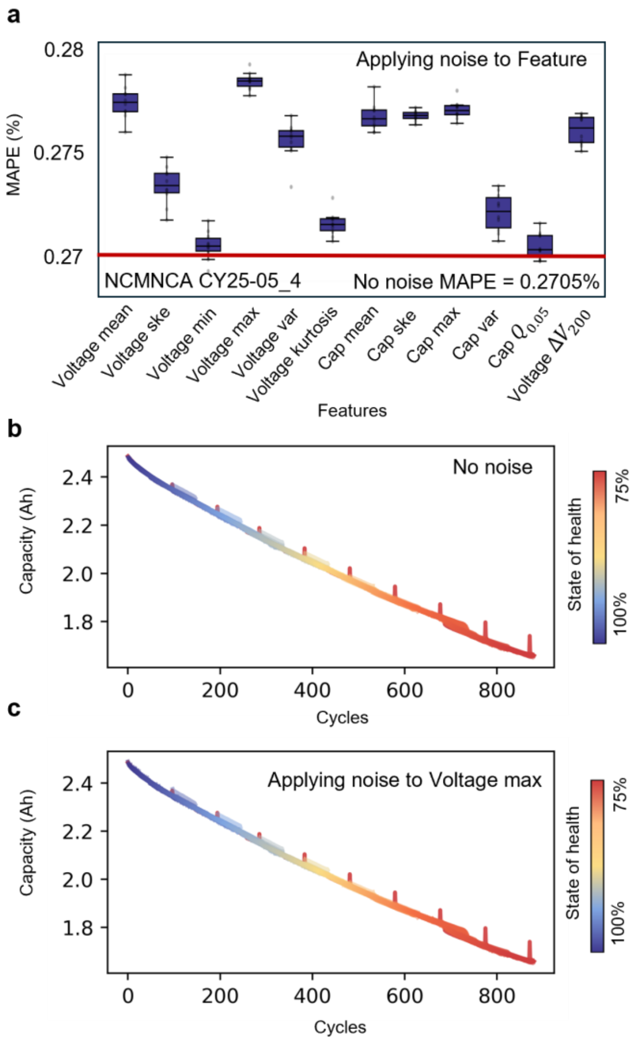

Supplementary Figure 27. SHAP Interpretability and Spearman Correlation Analysis Results

To quantify the influence of input features on the model's predictions and elucidate the underlying physical mechanisms, we employed SHapley Additive exPlanations (SHAP) analysis and Spearman's rank correlation analysis. As shown in Fig.(a), the SHAP analysis based on NCMNCA battery data reveals the differential contributions of the 12 physics-informed features to the final prediction outcome. Concurrently, Spearman's correlation analysis in Fig. (b) demonstrates that most features exhibit statistically significant correlations with SOH. These two analytical results corroborate each other, collectively confirming the robustness and reliability of the selected physical features in effectively characterizing the battery degradation state.

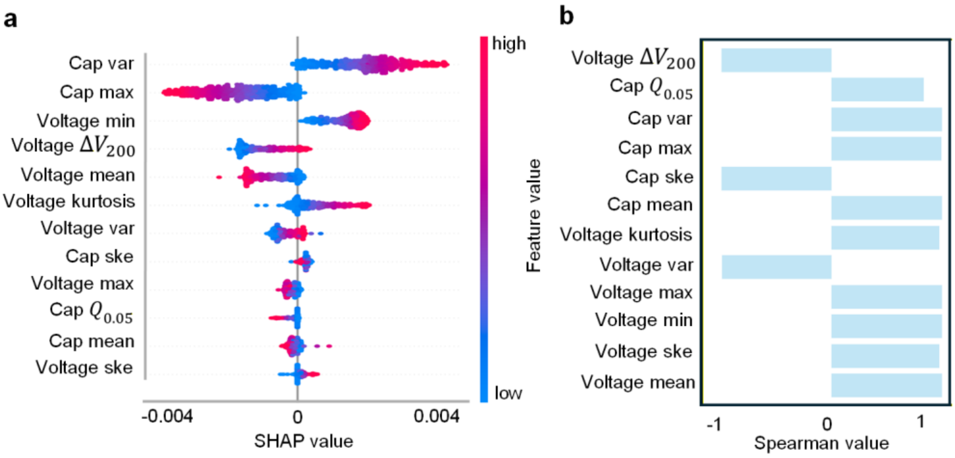

Supplementary Figure 28. Performance Analysis After Feature Reduction

The input of this paper consists of 12 features extracted from partial charging and relaxation voltage curves. Although 12 features may not seem many for modern machine learning methods, there may be issues with high correlation or redundancy among them. Based on SHAP and Spearman correlation analysis, we conducted further feature analysis and selection. Specifically, we removed 6 features that showed low correlation in both analyses to assess the impact of feature reduction on model performance. Each experiment was repeated 10 times to ensure rigor, with different colors representing the performance of the original model and the model with reduced feature count. The experimental results show that after removing the six low-correlation features, the model performance experienced a slight increase. This indicates that features with higher correlations have a more significant impact on the model's performance within the selected dataset. In deployment scenarios with limited computational resources, feature reduction can help strike an effective balance between performance and computational efficiency.

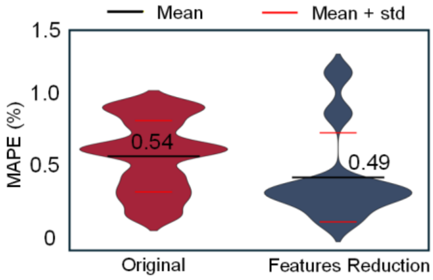

Supplementary Figure 29. Visualizations of the trend vectors across different cycles in various datasets

This section aims to provide a clearer explanation of the Trend vector we have defined, with visualizations of the trend vectors across different cycles in various datasets. The trend vector is a latent feature representation generated from field-acquired current cycle data, designed for predicting capacity degradation over multiple future cycles. In most conventional methods, the output of this vector is directly used as the prediction value. However, we argue that incorporating future load conditions is essential for meaningful real-world lifespan prediction, as future operating conditions significantly influence capacity degradation. In the original design, this vector reflects a preliminary prediction of future degradation based solely on the current health state, without accounting for the impact of future load conditions on degradation trajectories. The length L of the trend vector strictly corresponds to the number of prediction cycles. In the FORNN module, we integrate the trend vector with the planned future load on a cycle-by-cycle basis according to the prediction horizon, enabling the model to simultaneously achieve accurate assessment of the current state and embedding of the influence of future operating conditions. Different colors represent different cycle numbers.

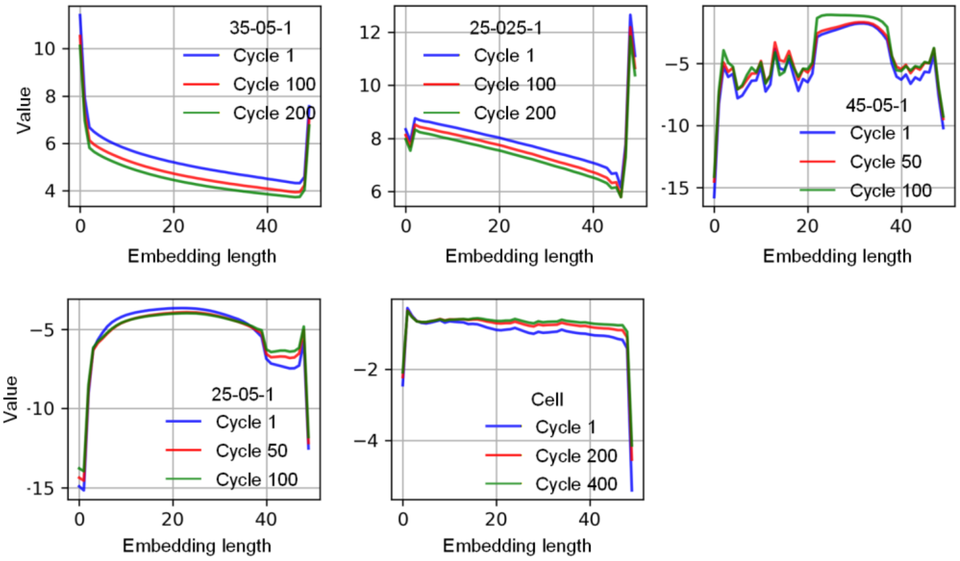

Supplementary Figure 30. Performance Comparison of the Proposed iMOE Model with and Without Historical Data.

We further investigate the applicability and performance of the iMOE model when directly predicting future degradation trajectories using existing maximum capacity data. Notably, since no within-cycle data is involved, we employ historical capacity to drive the weight allocation in our degradation router, treating capacity magnitude and degradation trajectory as classification criteria for expert network assignment. Each expert network directly outputs degradation trends based on historical cycles rather than capacity-voltage curves.

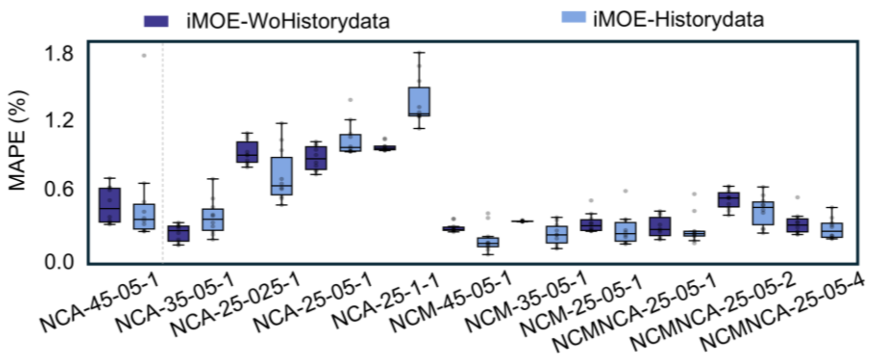

Supplementary Note 1. Data preprocessing and extraction procedures.

Here we show the data preprocessing and extraction procedures employed in our experimental study. Given the practical operating conditions, the presence of missing values and sampling errors in raw data represents a common scenario [1]. It should be emphasized that this research deliberately refrains from implementing sophisticated data preprocessing techniques to preserve the practical accessibility of field data, notwithstanding the potential compromise in model performance due to inherent and unavoidable measurement errors [2,3].

The data cleaning protocol involved the systematic removal of anomalous cycles meeting any of the following criteria: cycles exhibiting maximum capacity deviations exceeding 200mAh when compared to both preceding and subsequent cycles, cycles demonstrating zero capacity change during charging phases, and cycles showing abnormal current fluctuations during constant-current charging processes - all of which were identified as characteristic indicators of sampling irregularities. The final processed dataset was constructed by retaining only the continuous cycles that passed these quality control measures.

Let $Q_t \in \mathbb{R}^d$, represent the charging curve of the battery at cycle t, and $F_t \in \mathbb{R}^{12}$ denote the physical information features at cycle t, where d is the number of sampling points. The model predicts the maximum discharge capacity sequence for the next H cycles (see Supplementary Note 3 for specific prediction horizon). The load conditions for the next H cycles after cycle t are given by $C_t \in \mathbb{R}^{H\times 4}$.

Sample pairs (X, Y) are constructed using a sliding window approach:

$$\mathbf{X} = \begin{bmatrix} (Q_t, F_t, C_t) \\ (Q_{t+1}, F_{t+1}, C_{t+1}) \\ \vdots \\ (Q_{t+N-1}, F_{t+N-1}, C_{t+N-1}) \end{bmatrix} \tag{1}$$

$$\mathbf{Y} = \begin{bmatrix} y_{t+1} & y_{t+2} & \cdots & y_{t+H} \\ y_{t+2} & y_{t+3} & \cdots & y_{t+H+1} \\ \vdots & \vdots & \ddots & \vdots \\ y_{t+N} & y_{t+N+1} & \cdots & y_{t+N+H-1} \end{bmatrix} \tag{2}$$

where N represents the maximum number of cycles for the battery.

Supplementary Note 2. Feature extraction.

Inspired by Zhu et al., we extracted six statistical features from the relaxation voltage curve[4] (Max, Mean, Min, Var, Ske, Kur) and four from the charging capacity curve (Max, Mean, Var, Kur), with mathematical formulations detailed in Equation (3)-(8). Recognizing the strong correlation between increasing internal resistance and battery degradation during aging, we specifically designed two key physical-informative features to quantify lithium-ion activity loss: (1) $Q_{0.05}$, representing the charged capacity during a 0.05V voltage rise from the sampled SOC point; and (2) ΔV_{200}, denoting the voltage change corresponding to a 200mAh capacity increase from the same SOC reference. These dual features capture distinct degradation mechanism-$Q_{0.05}$reflects charge acceptance under mild polarization[5,6], while ΔV_{200} characterizes voltage hysteresis caused by capacity fade and electrode deterioration[7–9].

The twelve physical-informative features (six from each curve type) were normalized to F ∈ [0,1] for scale alignment, ensuring training stability. It should be noted that the input for driving the degradation gating router may include, but is not limited to, the features mentioned (e.g., IC curve peaks[10–12]). For the TPSL dataset, we exclusively used charging curve-derived features (excluding relaxation voltage characteristics) to demonstrate methodological generality. The standardization formula is as follows:

$$Maxima\ (Max) : x_{max} = max\{x_i\} \tag{3}$$

$$Mean\ (Mean) : \bar{x} = \frac{1}{n}\sum_{i-1}^{n} x_i \tag{4}$$

$$Minima\ (Min) : min\{x_i\} \tag{5}$$

$$Variance\ (Var) : \mu_2 = \frac{1}{n-1}\sum_{i=1}^{n} (x_i - \bar{x})^2 \tag{6}$$

$$Skewness\ (Ske) : \mu_3 = \frac{1}{n}\sum_{i=1}^{n} \left(\frac{x-\bar{x}}{\sqrt{\mu_2}}\right)^3 \tag{7}$$

$$Excess\ Kurtosis\ (Kur) : \mu_4 = \frac{1}{n}\sum_{i=1}^{n} \left(\frac{x-\bar{x}}{\sqrt{\mu_2}}\right)^4 - 3 \tag{8}$$

Supplementary Note 3. Selection of prediction horizons.

In practical applications, while extending the prediction horizon of degradation trajectories enhances model utility, excessive prediction spans may compromise accuracy as illustrated in Fig. 5d. Nevertheless, our proposed solution maintains robust performance even when predicting 150 cycles. To balance prediction accuracy with engineering practicality, this study adopts a 50-cycle prediction horizon as the benchmark (representing approximately 30% of a typical battery's median lifespan). Notably, for special operating conditions like NCA-25-1-1 with shorter cycle lives (~30 cycles), the prediction length is correspondingly reduced to 10 cycles to ensure reliability.

It should be emphasized that while existing literature reports longer prediction horizons[13], such methods typically require fixed sampling intervals and complete historical cycle data as input, a design that limits their deployment in dynamic operating conditions [14]. In contrast, our innovative approach requires only partial current cycle data as input and adapts to random initial SOC sampling strategies. This breakthrough not only significantly enhances real-world applicability but also provides technical assurance for end-user deployment convenience.

This design philosophy aligns perfectly with industry demands for “plug-and-play” prediction models, achieving an optimal compromise between prediction accuracy and data acquisition simplicity.

Supplementary Note 4. Implementation details of baselines and iMOE.

PatchTST[15] is a Transformer-based model utilizing patching technique. It enables effective pre-training and transfer learning across datasets. The source code is available at (https: //github.com/yuqinie98/PatchTST).

Informer[16] is a Transformer-based model specifically designed for long-sequence time-series forecasting. It introduces a ProbSparse self-attention mechanism to improve computational efficiency while maintaining prediction accuracy. The model also features a memory-efficient architecture that enables effective handling of large-scale datasets. The official implementation is available at (https://github.com/zhouhaoyi/Informer2020).

Both PatchTST and Informer were implemented according to their official source codes. For the Informer model, due to the unavailability of historical data for retired batteries, temporal information is reflected in the charging curve of the current cycle. We retained the encoder part and used an MLP as the decoder to serve as a predictor for the degradation trajectory. For the PatchTST model, the continuous sampling points of the charging curve were similarly treated as patch information. Where applicable, the baseline models and our proposed method used the same hyperparameters. We tuned the number of layers with reference to[13,17], the neuron number in hidden linear layers from[18], the weight decay in Adam optimizer within [0, 0.0001], and the number of attention heads for multi-head attention from [19,20]. The patch size was set to 3 based on the Q-V curve length[15]. The batch size was set to [8, 16, 32, 64, 128]. We applied for a learning rate of 0.001 across all datasets and selected the best-performing model on the validation set as our final model.

Supplementary Note 5. t-distributed stochastic neighbor embedding method.

The t-distributed stochastic neighbor embedding (t-SNE) method was employed to perform nonlinear dimensionality reduction on high-dimensional battery degradation features, enabling visualization of their low-dimensional distributions to reveal the relationship between degradation patterns at different cycle stages and expert weights. For the input feature matrix $\boldsymbol{X} \in \mathbb{R}^{\boldsymbol{n}\times\boldsymbol{d}}$ (where n represents the number of samples and d=5 corresponds to the number of experts), pairwise conditional probabilities between samples were calculated to construct the low-dimensional embedding that preserves these relationships in a visually interpretable space. This approach effectively captures the intrinsic clustering structure of battery degradation states while maintaining the relative associations between samples' expert weight distributions across various aging phases

$$p_{j|i} = \frac{\exp\left(-\| \mathbf{x}_i - \mathbf{x}_j \|^2 / 2\sigma_i^2\right)}{\sum_{k\neq i} \exp\left(-\| \mathbf{x}_i - \mathbf{x}_k \|^2 / 2\sigma_i^2\right)} \tag{9}$$

where σ_i is adaptively determined through perplexity (set to 30). By minimizing the KL divergence between the high-dimensional and low-dimensional distributions, the t-SNE algorithm preserves the local structure among samples in the original high-dimensional feature space while transforming global topological relationships into intuitively visible 2D distribution patterns, thereby clearly revealing the correlation characteristics between expert weights and battery degradation states.

$$\mathcal{L} = \sum_i KL(P_i \parallel Q_i) = \sum_i \sum_j p_{j|i} \log \frac{p_{j|i}}{q_{j|i}} \tag{10}$$

The similarity $q_{j|i}$ in the low-dimensional space is computed using a t-distribution:

$$q_{j|i} = \frac{\left(1 + \| \mathbf{y}_i - \mathbf{y}_j \|^2\right)^{-1}}{\sum_{k\neq l} \left(1 + \| \mathbf{y}_k - \mathbf{y}_l \|^2\right)^{-1}} \tag{11}$$

The t-SNE visualization plots for different datasets are provided in Supplementary Fig.17-19.

Supplementary Note 6. Deployment strategy for utilizing pre-trained models to make direct utilization decisions.

Here we demonstrate the deployment strategy for utilizing pre-trained models to make direct reuse decisions on retired batteries, as samples from different aging stages exhibit distinct differentiation in expert network weights. The trained model enables battery recyclers to predict degradation trajectories under uncertain operating conditions while making cost-effective utilization decisions, as the expert weights show strong correlation with degradation trends through iMOE's latent subspace clustering where different experts dominate prediction at respective stages. We implement a simple three-path classification system (high-efficiency secondary use for demanding applications like energy storage when Expert 1 dominates, direct recycling when Expert 5 dominates late degradation, and low-efficiency use for electronics in intermediate cases) based on single-cycle field data and future load condition inputs. The effectiveness of this expert weight-based classification is demonstrated in Fig. 4d, providing real-time decision-making with minimal data requirements while enabling adaptive resource allocation and cost optimization through precise battery health grading for practical large-scale retirement scenarios.

Supplementary Note 7. SHAP Interpretability Analysis

This section aims to elucidate the contribution of physics-informed features to the model outcomes through SHAP analysis. SHAP is an interpretability method grounded in cooperative game theory, designed to explain the predictions of machine learning models. Its core concept involves calculating the marginal contribution of each feature to individual prediction outcomes, thereby equitably allocating feature importance. SHAP values provide local interpretability, clearly demonstrating how each feature drives the model's prediction from the baseline value to the final output. This study employs SHAP analysis to reveal the decision-making basis of the constructed model and to identify the key factors and their direction of influence that drive the expert gating outcomes at different stages.

The SHAP value $\emptyset_i$ quantifies the contribution of feature i to the output $v(N)$, derived from its marginal contribution.

$$\emptyset_i = \sum_{S \in N/\{i\}} \frac{|S|!\,(n - |S| - 1)!}{n!} [v(S \cup \{i\}) - v(S)] \tag{12}$$

where S is a subset of the full feature set N, and $|S|$ indicates the size (number of features) of the subset. The function is the payoff function for the subset S.

The sum of the SHAP values of all features equals the difference between the predicted value for the sample and the baseline value (the expected prediction value of the model)

$$g(z') = \emptyset_0 + \sum_{i=1}^{M} \emptyset_i z_i' , \tag{13}$$

Here, $\emptyset_0$ represents the baseline prediction of the model, $\emptyset_i$ denotes the SHAP value of feature, and M indicates the total number of features.

Supplementary Note 8. Explanation map.

We define a vector Trend to represent the output of the AMDP module and visualize the hidden outputs of input samples across the full lifecycle to explain the performance improvement of iMOE. In Fig.4g, using the NCA-35-05-1 battery, we normalize each value of the Trend vector across different samples for clearer interpretation, thereby demonstrating how the model internally processes samples at different aging stages and clarifying its contribution to degradation trajectory prediction. For the input X of the iMOE model, the Trend is obtained through the AMDP module.

$$\mathrm{Trend} = \mathrm{AMDP}(\mathrm{X}) \tag{14}$$

$\mathrm{Trend} \in \mathbb{R}^{\mathrm{n} \times \mathrm{L}}$, where n denotes the number of samples and L represents the prediction length.

Supplementary Note 9. Degradation Trajectory Prediction Method (Applicable to Scenarios with Complete Historical Data and Consistent Operating Conditions).

The experiments in this section focus on predicting the future degradation trajectory of retired batteries under the condition that historical data is available and future operating conditions remain unchanged. However, this scenario imposes stringent requirements on historical data collection and future usage predictions, which often deviate from real-world applications [21]. To demonstrate that relatively simple methods can achieve satisfactory prediction results, we employ polynomial fitting and a three-layer MLP network. For the polynomial fitting method, given a total cycle count N for the battery, we define a sliding window of size w with a step sizes. For the t-th window:

$$\mathcal{W}_t = \{y_t, y_{t+1}, \ldots, y_{t+\omega-1}\}, t \in [0, N-\omega-s] \tag{15}$$

where $y_i$represents the capacity at the i-th cycle. For each sliding window, we construct a polynomial regression model to capture the capacity degradation trend within that specific segment of the battery's lifespan. This approach models the relationship between cycle number and capacity using a polynomial function, allowing for flexible fitting of nonlinear degradation patterns observed in different phases of the battery's aging process.

$$\hat{y}(x) = \beta_0 + \beta_1 x + \beta_2 x^2 + \cdots + \beta_d x^d \tag{16}$$

where $\mathrm{x} \in [\mathrm{t}, \mathrm{t}+\omega-1]$ represents cycle index, the model parameters $\widehat{\boldsymbol{\beta}} \in \mathbb{R}^{\mathrm{d}+1}$ are determined via least squares:

$$\widehat{\boldsymbol{\beta}} = \arg \min_{\boldsymbol{\beta}} \sum_{i=t}^{t+\omega-1} (y_i - \hat{y}(i))^2 \tag{17}$$

The fitted model is used to predict the next S cycles:

$$\hat{y}_{t+\omega+k} = \hat{\beta}_0 + \hat{\beta}_1(t+\omega+k) + \cdots + \hat{\beta}_d(t+\omega+k)^d, k = 0, \ldots, s-1 \tag{18}$$

We employ a cubic polynomial (d=3) for fitting, with a window size ω=50 cycles, prediction step s=50, and sliding window stride of 1. For the MLP, we adopt an end-to-end processing method that models the prediction of future degradation trajectories from historical capacity data as a time series forecasting problem. Given a capacity sequence $Q = \{q_1, q_2, \ldots, q_T\}$, with defined window size w and prediction steps, the k-th sample pair is expressed as:

$$\begin{cases} \mathbf{X}_k = [q_{k\delta}, q_{k\delta+1}, \ldots, q_{k\delta+w-1}] \\ \mathbf{Y}_k = [q_{k\delta+w}, q_{k\delta+w+1}, \ldots, q_{k\delta+w+s-1} \end{cases} \tag{19}$$

Where $k \in \left[0, \left\lfloor \frac{T-w-s}{\delta} \right\rfloor\right]$. A three-layer MLP network is designed：

$$\begin{aligned} \mathbf{h}_1 &= \mathrm{ReLU}\ (\mathbf{W}_1 \mathbf{X} + \mathbf{b}_1) \\ \mathbf{h}_2 &= \mathrm{ReLU}\ (\mathbf{W}_2 \mathbf{h}_1 + \mathbf{b}_2) \\ \hat{Y} &= \mathbf{W}_3 \mathbf{h}_2 + \mathbf{b}_3 \end{aligned} \tag{20}$$

Where weight matrices $\mathbf{W}_1 \in \mathbb{R}^{128\times w}, \mathbf{W}_2 \in \mathbb{R}^{64\times 128}, \mathbf{W}_3 \in \mathbb{R}^{s\times 64}$. In our experiments, we set the window size $\omega = 10$ cycles, prediction step s=50, and sliding window stride to 1.

Supplementary Note 10. The proposed iMOE framework for degradation trajectory prediction using historical capacity data.

In certain scenarios, battery recyclers may obtain partial historical maximum capacity data prior to battery retirement, which enables direct recycling without additional testing. Here, we further investigate the applicability and performance of the iMOE model when directly predicting future degradation trajectories using existing maximum capacity data. Notably, since no within-cycle data is involved, we utilize historical capacity to drive the weight allocation in our degradation router, treating capacity magnitude and degradation trajectory as classification criteria for expert network assignment. Each expert network directly outputs degradation trends based on historical cycles rather than capacity-voltage curves. The historical data are decomposed via a feed-forward network (FFN) and fed into a noisy degradation router:

$$H(Q) = \mathbf{Q} \cdot \mathbf{W}_g + \psi \cdot \mathrm{Softplus}(Q \cdot \mathbf{W}_{\mathrm{noise}}) \tag{21}$$

Here, $\mathbf{Q}$ represents the historical cycle capacity data. The expert network weights $G(Q)$are then derived from $\mathbf{Q}$using the same methodology described in the Methods section. These adaptively integrated expert weights generate preliminary degradation trend predictions.

$$\boldsymbol{Trend_i} = \sum_{j=0}^{E} \mathbf{G}\,(\boldsymbol{Q}_{\mathbf{i}}) \cdot \mathrm{Predictor}_j(\mathbf{Q}_{\mathbf{i}}) \tag{22}$$

The final degradation trajectory prediction is achieved by progressively incorporating future operating conditions using the identical FORNN architecture. Model prediction results are shown in Supplementary Fig. 30.

Supplementary Note 11. Data reduction strategy.

The availability of retired battery data significantly hinders the transition of data-driven approaches from laboratory to real-world deployment [21–23]. This section demonstrates how to evaluate the robustness of the proposed model under data-constrained conditions. We employ a progressive data reduction strategy: while keeping the test set unchanged, we systematically reduce the number of batteries in the training set for each specific operating condition to 20%, 40%, 60%, and 80% of the original training set (the complete training set contains 78 batteries, with the 20% subset corresponding to 21 batteries, following the rounding-up principle). This approach accurately simulates the data acquisition limitations commonly encountered in practical applications.

Supplementary Table 1. Technical Specifications of UL Dataset.

The table presents the material types, operating conditions, nominal capacities, cutoff voltages, and number of batteries used in our experimental dataset. All batteries in this study underwent identical load cycling throughout their full lifecycle until aging. The dataset is designated as the UL Dataset[4], and we adhere to the naming convention established by the data providers. For instance, the notation "NCA-45-05-1" refers to an NCA battery tested at 45°C with 0.5C CCCV charging and 1C discharging conditions, with similar naming logic applying to other cases. For the NCM + NCA battery, the electrochemical impedance is conducted every 50 cycles at full charge in a range of 10 kHz to 0.01 Hz (6 data points per decade of frequency) with a sinusoidal amplitude of 250 mA. 60 min are set at the open circuit voltage before the electrochemical impedance tests. The UL Dataset encompasses 3 material types, 11 operating conditions, and 130 batteries in total. Notably, for each sample, we utilize only the current cycle's data without requiring any historical cycle information.

Cell types	Material	Working conditions			Nominal capacity (Ah)	Cut-off voltage (V)	Number of cells
		Charge C rate	Discharge C rate	Temperature (° C)			
NCA	$Li_{0.86}Ni_{0.86}Co_{0.11}Al_{0.03}O_2$ / Graphite	0.5	1	25	3.5	2.65-4.2	66
		0.5	1	35			
		0.5	1	45			
		1	1	25			
		0.25	1	25			
NCM+NCA	42 (3) wt.% $Li(NiCoMn)O_2$ blended with 58 (3) wt.% $Li(NiCoAl)O_2$/ Graphite	0.5	1	25	2.5	2.5–4.2	9
		0.5	2	25			
		0.5	4	25			
NCM	$LiNi_{0.83}Co_{0.11}Mn_{0.07}O_2$/ Graphite	0.5	1	25	3.5	2.5-4.2	55
		0.5	1	35			
		0.5	1	45			

Supplementary Table 2. Technical Specifications of LSD Dataset.

The table presents the materials, operating conditions, nominal capacities, cutoff voltages, and number of batteries used in our experimental dataset. During phase 1, as the SOH of the batteries declined from 100% to 80%, 86 cells were divided into 16 groups, with each group subjected to a distinct charge–discharge protocol. In phase II, as the SOH further decreased from 80% to 50%, all 86 cells were cycled under a unified protocol. The dataset is designated as the LSD Dataset.

In the phase 2 dataset, although all batteries were operated under identical cycling conditions, differences in the loading conditions during their first-life usage led to varying impacts on internal degradation mechanisms, resulting in significantly divergent degradation trajectories during the second-life phase. This phenomenon highlights the profound influence of historical usage conditions on the long-term health evolution of batteries and provides an ideal validation scenario for evaluating the model's predictive capability under unknown historical conditions and deep degradation states. We utilized only the second-phase dataset to simulate the model's ability to predict degradation trajectories of deeply aged retired batteries in deployment scenarios where historical data are unavailable.

Cell types	Material	Working conditions			Nominal capacity (Ah)	Cut-off voltage (V)	Number of cells
		Charge C rate	Discharge C rate	Temperature (° C)			
Phase 1	$LiCoO_2$ and $LiNi_{0.5}Co_{0.2}Mn_{0.3}O_2$ / graphite anodes	0.5	1	25	2.4	3.0-4.2	8
		0.5	1	25			8
		0.5	1	25			6
		1	1	25			8
		1.5	1	25			8
		2	1	25			4
		0.5	2	25			4
		0.5	3	25			4
		1	1	25			4
		1	1	25			4
		1.5	1	25			4
		1.5	1	25			4
		2	3	25			4
		0.5	0.2	25			3
		0.5	0.5	25			7
		0.5	2	25			4
Phase 2		0.5	1	25			86

Supplementary Table 3. Technical Specifications of TPSL Dataset.

The table presents the materials, operating conditions, nominal capacities, cutoff voltages, and number of batteries used in our experimental dataset. After the initial 20 cycles, the load conditions were modified for these batteries, with 22 batteries undergoing different constant operating condition cycles and 55 batteries subjected to random operating condition cycles. The dataset is designated as the TPSL Dataset[24].

In this study, "TPSL-Random" refers to batteries operating under random conditions during their second-life usage, while "TPSL-Fixed" indicates batteries operating under fixed conditions during their second-life usage. The TPSL Dataset encompasses a total of 66 second-life operating conditions (55 random and 11 fixed) across 77 batteries. In the TPSL dataset, the charging protocol was changed from the CCCV method used during the first life to the CC method employed in the second-life stage. This shift in operating conditions resulted in a significant sudden capacity drop between the cycles adjacent to the transition point. In the TPSL-Fixed dataset, batteries under different fixed conditions exhibited significantly divergent degradation trajectories. In the TPSL-Random dataset, the random operating conditions resulted in notable maximum capacity differences even between adjacent cycles. This further underscores the necessity of incorporating future operating conditions as conditional input. Notably, for each sample, we utilize only the current cycle's data without requiring any historical cycle information.

Cell types	Material	Working conditions			Nominal capacity (Ah)	Cut-off voltage (V)	Number of cells
		Charge C rate	Discharge C rate	Temperature (° C)			
NCM	$LiNi_{0.5}Co_{0.2}Mn_{0.3}O_2$ / graphite anodes	2	1	25	2.4	3.0-4.2	3
		3	1	25			3
		1	2	25			4
		2	2	25			3
		3	2	25			3
		2	3	25			3
		3	3	25			3
		Random current (1C~3C)	3	25			55

Supplementary Table 4. The model core configuration.

Here we show the hyperparameter configuration for our experiments. While more sophisticated training schemes and precise hyperparameter tuning might yield better results, we maintained identical hyperparameters across all datasets and operating conditions, except for the training length (set to 10 for NCA-25-1-1 dataset and 50 for others) to ensure valid experimental verification through maximally simplified configurations.

Level	Layer name	Parameter
	Expert Number	5
	Topk	2
AMDP	Degradation Router	$(I_S, O_S) = (12,5)$
	α	10
	Predictor	$(I_S, O_S) = (50,50)$
	hidden dim	64
FORNN	FFN	$(I_S, O_S) = (64,1)$
	Dropout	0.05
	Activation function	ReLu

Supplementary Table 5. The performance of the model in four different usage scenarios.

Here, we demonstrate the predictive performance of the iMOE model under three distinct scenarios: UL representing the full lifecycle under constant operating conditions, LSD representing the deep degradation dataset, and TPSL corresponding to repurposed usage with varying operating conditions (specific naming conventions are detailed in Supplementary Table 1). To ensure experimental rigor, all results represent averages from 10 randomized trials. Notably, the models exclusively utilized partial cycle data collected in situ as input to predict 50-cycle capacity degradation trajectories under uncertain future operating conditions.

Error Metric	MAPE (%)	RMSE (%)	MAE (%)
UL	0.520852	1.878831	1.404282
TPSL-Arbitrary	2.957535	4.277484	3.326660
TPSL-Fixed	2.806381	5.522538	3.857384
LSD	1.809982	3.655229	2.805560

Supplementary Table 6. Classification results based on expert-weighted evaluation for batteries at different SOH retirement scenarios.

Condition	SOH	Total numbers	Battery Classification			Confidence Level
			Excellent	Qualified	Scrap	
NCACY45-05_1	95	28	27	1	0	96.4%
	85	28	19	3	3	/
	75	28	1	1	26	92.9%
NCACY25-05_1	95	19	19	0	0	100%
	85	19	5	5	9	/
	75	19	0	0	19	100%
NCACY25-1_1	95	9	9	0	0	100%
	85	9	0	4	5	/
	75	9	0	0	9	100%
NCACY25-025_1	95	7	6	1	0	85.7%
	85	7	0	5	2	/
	75	6	0	2	4	66.7%
NCACY35-05_1	95	3	3	0	0	100%
	85	3	2	0	1	/
	75	2	0	0	2	100%
NCMCY45-05_1	95	28	19	9	0	67.9%
	85	28	24	3	1	/
	75	23	0	0	23	100%
NCMCY35-05_1	95	4	4	0	0	100%
	85	4	4	0	0	/
	75	4	0	0	4	100%
NCMCY25-05_1	95	23	23	0	0	100%
	85	23	5	6	12	/
	75	7	0	0	7	100%
NCMNCACY25-05_1	95	3	3	0	0	100%
	85	3	0	3	0	/
	75	3	0	0	3	100%
NCMNCACY25-05_2	95	3	3	0	0	100%
	85	3	3	0	0	/
	75	3	0	0	3	100%
NCMNCACY25-05_4	95	3	3	0	0	100%
	85	3	3	0	0	/
	75	3	0	0	3	100%

Supplementary Table 7. Model performance under 80% training data condition.

Here we address how limited data availability hinders the transition of data-driven approaches from laboratory to real-world deployment by evaluating iMOE, PatchTST and Informer performance under constrained training data conditions. As a case study, we systematically reduce the training data to 80% of the original experimental dataset to examine the relationship between training data volume and model performance. All results represent average MAPE (%) from 10 randomized trials, with models exclusively using partial cycle data collected in situ to predict 50-cycle capacity degradation trajectories under uncertain future operating conditions.

Model	iMOE	PatchTST	Informer
NCACY45-05_1	0.38524	1.30522	1.32668
NCACY35-05_1	0.54664	0.41313	0.65608
NCACY25-025_1	0.99175	1.46143	1.20459
NCACY25-05_1	1.1911	1.09639	1.13361
NCACY25-1_1	1.27391	1.28571	1.30616
NCMCY45-05_1	0.35657	0.36611	0.87559
NCMCY35-05_1	0.45163	0.35729	0.63922
NCMCY25-05_1	0.47977	0.30736	0.64768
NCMNCACY25-05_1	0.3374	0.37809	0.50637
NCMNCACY25-05_2	0.52637	0.52904	0.5344
NCMNCACY25-05_4	0.35185	0.40742	0.57785
TPSL-Arbitrary	3.214	16.29637	16.3254
TPSL-Fixed	3.24584	30.6155	31.88632

Supplementary Table 8. Model performance under 60% training data condition.

Here we address how limited data availability hinders the transition of data-driven approaches from laboratory to real-world deployment by evaluating iMOE, PatchTST and Informer performance under constrained training data conditions. As a case study, we systematically reduce the training data to 60% of the original experimental dataset to examine the relationship between training data volume and model performance. All results represent averaged MAPE (%) from 10 randomized trials, with models exclusively using partial cycle data collected in situ to predict 50-cycle capacity degradation trajectories under uncertain future operating conditions.

Model	iMOE	PatchTST	Informer
NCACY45-05_1	0.48513	1.37203	2.67657
NCACY35-05_1	0.54664	0.41313	2.47614
NCACY25-025_1	1.05483	1.70332	4.17579
NCACY25-05_1	1.18969	1.12744	2.2514
NCACY25-1_1	1.54336	1.35142	1.34329
NCMCY45-05_1	0.38032	1.1135	1.16151
NCMCY35-05_1	0.45163	0.35549	0.73698
NCMCY25-05_1	0.49066	0.3395	1.46621
NCMNCACY25-05_1	0.3374	0.37809	1.47611
NCMNCACY25-05_2	0.52637	0.52904	1.47845
NCMNCACY25-05_4	0.35185	0.40985	1.02613
TPSL-Arbitrary	3.20795	16.40015	16.16911
TPSL-Fixed	3.9755	30.95967	32.02418

Supplementary Table 9. Model performance under 40% training data condition.

Here we address how limited data availability hinders the transition of data-driven approaches from laboratory to real-world deployment by evaluating iMOE, PatchTST and Informer performance under constrained training data conditions. As a case study, we systematically reduce the training data to 40% of the original experimental dataset to examine the relationship between training data volume and model performance. All results represent average MAPE (%) from 10 randomized trials, with models exclusively using partial cycle data collected in situ to predict 50-cycle capacity degradation trajectories under uncertain future operating conditions.

Model	iMOE	PatchTST	Informer
NCACY45-05_1	0.5128	1.42162	2.66275
NCACY35-05_1	0.57415	0.57858	2.79688
NCACY25-025_1	1.32399	1.65556	3.94724
NCACY25-05_1	1.20611	1.11754	2.68264
NCACY25-1_1	1.69018	1.50498	1.59012
NCMCY45-05_1	0.44771	0.98612	1.36852
NCMCY35-05_1	0.48234	0.43736	0.70738
NCMCY25-05_1	0.55269	0.3646	1.82602
NCMNCACY25-05_1	0.36781	0.53223	1.67119
NCMNCACY25-05_2	0.68287	0.61551	1.64966
NCMNCACY25-05_4	0.3993	0.54425	1.0907
TPSL-Arbitrary	3.39716	16.45436	16.31379
TPSL-Fixed	4.31718	31.22801	33.0205

Supplementary Table 10. Predicting the performance of different models over the next 30 cycles.

Here we examine the relationship between prediction horizon length and model performance in practical deployment scenarios. We systematically evaluate degradation trajectory predictions under uncertain future operating conditions with 30-cycle prediction lengths, while maintaining the model's input as partial current-cycle data. Notably, as previously mentioned, the NCACY25-1_1 dataset (with an average lifespan of approximately 30 cycles) retains a prediction length of 10 cycles. All results represent average MAPE (%) from 10 randomized trials to ensure experimental rigor.

Model	iMOE	PatchTST	Informer
NCACY45-05_1	0.34547	1.18713	1.35917
NCACY35-05_1	0.41449	0.41208	0.59813
NCACY25-025_1	1.16528	1.42827	1.06976
NCACY25-05_1	0.92025	0.9871	1.09392
NCACY25-1_1	0.97902	1.55313	1.96347
NCMCY45-05_1	0.32087	0.23197	0.66038
NCMCY35-05_1	0.6524	0.37717	0.64623
NCMCY25-05_1	0.3936	0.30995	0.66665
NCMNCACY25-05_1	0.38524	0.6007	0.58618
NCMNCACY25-05_2	0.56414	0.6626	0.49081
NCMNCACY25-05_4	0.42391	0.482	0.48673
TPSL-Arbitrary	2.18022	15.18396	15.20092
TPSL-Fixed	1.74537	19.34302	19.8816

Supplementary Table 11. Predicting the performance of different models over the next 80 cycles.

Here we examine the relationship between prediction horizon length and model performance in practical deployment scenarios. We systematically evaluate degradation trajectory predictions under uncertain future operating conditions with 80-cycle prediction lengths, while maintaining the model's input as partial current-cycle data. Notably, as previously mentioned, the NCACY25-1_1 dataset (with an average lifespan of approximately 30 cycles) retains a prediction length of 10 cycles. For the TPSL dataset (maximum 120 cycles), the 80-cycle prediction effectively constitutes an early-life prediction task. All results represent average MAPE (%) from 10 randomized trials to ensure experimental rigor.

Model	iMOE	PatchTST	Informer
NCACY45-05_1	0.5128	1.42162	2.66275
NCACY35-05_1	0.57415	0.57858	2.79688
NCACY25-025_1	1.32399	1.65556	3.94724
NCACY25-05_1	1.20611	1.11754	2.68264
NCACY25-1_1	1.69018	1.50498	1.59012
NCMCY45-05_1	0.44771	0.98612	1.36852
NCMCY35-05_1	0.48234	0.43736	0.70738
NCMCY25-05_1	0.55269	0.3646	1.82602
NCMNCACY25-05_1	0.36781	0.53223	1.67119
NCMNCACY25-05_2	0.68287	0.61551	1.64966
NCMNCACY25-05_4	0.3993	0.54425	1.0907
TPSL-Arbitrary	3.39716	16.45436	16.31379
TPSL-Fixed	4.31718	31.22801	33.0205

Supplementary Table 12. Predicting the performance of different models over the next 100 cycles**.**

Here we examine the relationship between prediction horizon length and model performance in practical deployment scenarios. We systematically evaluate degradation trajectory predictions under uncertain future operating conditions with 100 cycles prediction lengths, while maintaining the model's input as partial current-cycle data. Notably, as previously mentioned, the NCACY25-1_1 dataset (with an average lifespan of approximately 30 cycles) retains a prediction length of 10 cycles. For the TPSL dataset (maximum 120 cycles), the 100-cycle prediction effectively constitutes an early-life prediction task. All results represent average MAPE (%) from 10 randomized trials to ensure experimental rigor.

Model	iMOE	PatchTST	Informer
NCACY45-05_1	0.54324	0.95772	1.13189
NCACY35-05_1	0.53951	0.51083	0.60636
NCACY25-025_1	1.49899	1.51116	1.27905
NCACY25-05_1	0.98466	1.00232	1.06487
NCACY25-1_1	1.22211	1.55313	1.96347
NCMCY45-05_1	0.39807	0.1943	0.72192
NCMCY35-05_1	0.44048	0.36232	0.64616
NCMCY25-05_1	0.4945	0.31077	0.55818
NCMNCACY25-05_1	0.31185	0.3969	0.48392
NCMNCACY25-05_2	0.60576	0.55978	0.49276
NCMNCACY25-05_4	0.49869	0.49689	0.55327
TPSL-Arbitrary	5.31326	23.88182	23.88243
TPSL-Fixed	6.25851	72.2825	70.19593

Supplementary References

1. Tao, S. et al. Generative learning assisted state-of-health estimation for sustainable battery recycling with random retirement conditions. Nat Commun 15, 10154 (2024).
2. Wang, Z. et al. Battery health diagnostics: Bridging the gap between academia and industry. eTransportation 19, 100309 (2024).
3. Innocenti, A., Beringer, S. & Passerini, S. Cost and performance analysis as a valuable tool for battery material research. Nat Rev Mater 9, 347–357 (2024).
4. Zhu, J. et al. Data-driven capacity estimation of commercial lithium-ion batteries from voltage relaxation. Nat Commun 13, 2261 (2022).
5. Pinson, M. B. & Bazant, M. Z. Theory of SEI Formation in Rechargeable Batteries: Capacity Fade, Accelerated Aging and Lifetime Prediction. J. Electrochem. Soc. 160, A243–A250 (2013).
6. Xiong, R., Wang, P., Jia, Y., Shen, W. & Sun, F. Multi-factor aging in Lithium Iron phosphate batteries: Mechanisms and insights. Appl. Energy 382, 125250 (2025).
7. Che, Y., Hu, X., Lin, X., Guo, J. & Teodorescu, R. Health prognostics for lithium-ion batteries: mechanisms, methods, and prospects. Energy Environ. Sci. 16, 338–371 (2023).
8. Wang, J. et al. Degradation of lithium ion batteries employing graphite negatives and nickel–cobalt–manganese oxide + spinel manganese oxide positives: Part 1, aging mechanisms and life estimation. J. Power Sources 269, 937–948 (2014).
9. Birkl, C. R., Roberts, M. R., McTurk, E., Bruce, P. G. & Howey, D. A. Degradation diagnostics for lithium ion cells. J. Power Sources 341, 373–386 (2017).
10. Ye, J., Xie, Q., Lin, M. & Wu, J. A method for estimating the state of health of lithium-ion batteries based on physics-informed neural network. Energy 294, 130828 (2024).
11. Xiong, R. et al. A data-driven method for extracting aging features to accurately predict the battery health. Energy Storage Mater. 57, 460–470 (2023).
12. Roman, D., Saxena, S., Robu, V., Pecht, M. & Flynn, D. Machine learning pipeline for battery state-of-health estimation. Nat Mach Intell 3, 447–456 (2021).
13. Tan, R. et al. Forecasting battery degradation trajectory under domain shift with domain generalization. Energy Storage Mater. 72, 103725 (2024).
14. Hsu, C.-W., Xiong, R., Chen, N.-Y., Li, J. & Tsou, N.-T. Deep neural network battery life and voltage prediction by using data of one cycle only. Appl. Energy 306, 118134 (2022).
15. Nie, Y., Nguyen, N. H., Sinthong, P. & Kalagnanam, J. A Time Series is Worth 64 Words: Long-term Forecasting with Transformers. Preprint at https://doi.org/10.48550/arXiv.2211.14730 (2023).
16. Zhou, H. et al. Informer: Beyond Efficient Transformer for Long Sequence Time-Series Forecasting. AAAI 35, 11106–11115 (2021).
17. Hu, Y., Liu, P., Zhu, P., Cheng, D. & Dai, T. Adaptive Multi-Scale Decomposition Framework for Time Series Forecasting. Preprint at https://doi.org/10.48550/arXiv.2406.03751 (2024).
18. Strange, C. & Dos Reis, G. Prediction of future capacity and internal resistance of Li-ion cells from one cycle of input data. Energy and AI 5, 100097 (2021).
19. Shi, X. et al. Time-MoE: Billion-Scale Time Series Foundation Models with Mixture of Experts. Preprint at https://doi.org/10.48550/arXiv.2409.16040 (2024).
20. Wang, Y. et al. TimeXer: Empowering Transformers for Time Series Forecasting with Exogenous Variables. Preprint at https://doi.org/10.48550/arXiv.2402.19072 (2024).
21. Zhao, M., Zhang, Y. & Wang, H. Battery degradation stage detection and life prediction without accessing

historical operating data. Energy Storage Mater. 69, 103441 (2024).

22. Wu, W., Lin, B., Xie, C., Elliott, R. J. R. & Radcliffe, J. Does energy storage provide a profitable second life for electric vehicle batteries? Energy Economics 92, 105010 (2020).

23. Duffner, F., Wentker, M., Greenwood, M. & Leker, J. Battery cost modeling: A review and directions for future research. Renew. Sustain. Energy Rev. 127, 109872 (2020).

24. Lu, J. et al. Battery degradation prediction against uncertain future conditions with recurrent neural network enabled deep learning. Energy Storage Mater. 50, 139–151 (2022).
